# Sustainable Cellulose Enables Blue Energy Toward Osmotic Energy Conversion

**DOI:** 10.1007/s40820-026-02213-9

**Published:** 2026-05-20

**Authors:** Yingchao Wang, Jianping Shi, Qianhong Zhang, Hui Wu, Qingxian Miao, Liulian Huang, Lihui Chen, Yonghao Ni, Jianguo Li

**Affiliations:** 1 College of Material Engineering, National Forestry and Grassland Administration Key Laboratory of Plant Fiber Functional Materials, Fujian Agriculture and Forestry University, Fuzhou, 350002 People’s Republic of China; 2 Department of Chemical Engineering, University of New Brunswick, Fredericton, NB E3B5A3 Canada

**Keywords:** Cellulose, Ion-selective transport, Osmotic energy conversion, Nanofluidic membrane, Structural and chemical distinction

## Abstract

The development of osmotic energy technologies offers a sustainable and renewable pathway to address global energy shortages and environmental challenges. Cellulose-based membranes have been increasingly recognized for their remarkable potential in osmotic energy conversion, owing to their intrinsic ion-selective transport properties, structural and chemical tunability. This outstanding performance is primarily attributed to the renewable origin, versatile surface chemistry, and mechanical robustness of cellulose, which collectively facilitate the design of sustainable and durable ion-conducting membranes. This review highlights recent advances in the design and application of cellulose-based membranes for salinity-gradient energy harvesting, with an emphasis on material composition, nanoscale structural engineering, surface functionalization, and optimization of the ion transport approach. Despite these advances, key challenges that hinder further performance enhancement are identified and critically discussed, along with potential strategies for practical large-scale implementation. Furthermore, recent advances in nanoarchitectonic design and chemical functionalization have demonstrated significant improvements in power density, long-term operational stability, and overall membrane performance under diverse salinity and environmental conditions, underscoring the promise of cellulose-based membranes for next-generation blue energy technologies.

## Highlights

- This review highlights the design and application of cellulose-based membranes for salinity-gradient energy harvesting.
- Material composition, nanoscale structural engineering, surface functionalization, and ion-transport regulation strategies are discussed.
- Stability, robustness, and scalability are systematically evaluated, and emerging strategies directly relevant to practical applications are summarized.

## Graphical abstract

**Figure:**
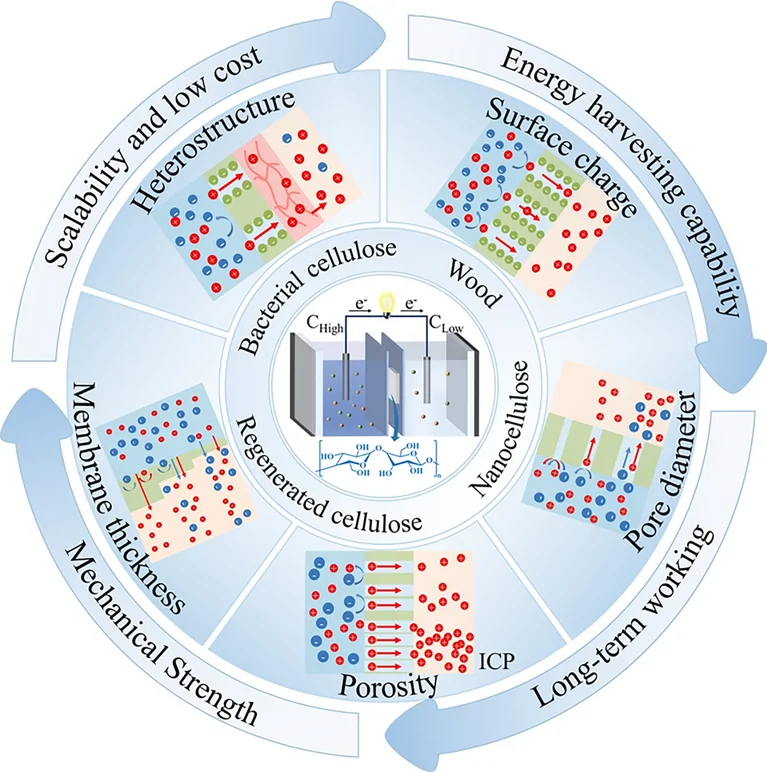

## Introduction

Electricity underpins modern industry and daily life. The rapid expansion of energy-intensive sectors, particularly artificial intelligence (AI) and large-scale data centers, further accelerates this demand. For example, a 1 GW-class AI computing center running continuously for one year consumes on the order of 8.76 TWh of electricity, comparable to the annual consumption of over one million households in developed countries [1]. At present, fossil fuels still dominate global power generation, but their finite reserves and severe environmental impacts have made the development of sustainable alternatives an urgent priority. Renewable technologies such as solar, wind, and hydropower have substantially reduced carbon emissions and are widely recognized as key pillars for achieving carbon neutrality [2–5]. However, their output is inherently intermittent and constrained by geographic distribution, weather conditions, and seasonal fluctuations. To complement these variable resources, researchers are actively exploring continuous, geographically ubiquitous clean energy sources, including geothermal, nuclear fusion, and osmotic (salinity-gradient) energy.

Osmotic energy, or “blue energy”, refers to the conversion of the Gibbs free energy released when freshwater mixes with seawater into electricity using semipermeable or ion-exchange membranes [6, 7]. The theoretical global power potential associated with river discharge into the oceans is estimated to be ~ 2000 GW, of which ~ 980 GW is considered technically recoverable with current technologies [8]. Anthropogenic wastewater discharges could contribute an additional ~ 18 GW [9]. If efficiently harvested, this recoverable osmotic energy could support on the order of thousands of 1 GW-class facilities, underscoring the strategic importance of salinity-gradient energy for relieving global energy pressure and advancing environmental sustainability.

In membrane-based osmotic energy conversion systems, particularly reverse electrodialysis (RED), the fundamental mechanism relies on the selective transport of cations and anions through ion-exchange membranes to convert the chemical potential difference between solutions with different salinities into electrical energy [10–13]. Under a salinity gradient, cation-exchange membranes (CEMs) and anion-exchange membranes (AEMs) preferentially transport counter-ions while excluding co-ions [10, 11, 14]. The ion selectivity primarily arises from the Donnan exclusion effect associated with the fixed charges within the membrane matrix. In the presence of a concentration gradient, this selective ion transport gives rise to a transmembrane potential, namely the membrane potential, in accordance with the Nernst equation [12, 15–17]. In a typical RED stack, CEMs and AEMs are arranged alternately, allowing the membrane potentials generated by individual membrane units to accumulate spatially and produce a macroscopic potential difference. The potential difference drives directional ion migration under the coupled action of the concentration gradient and electric field, thereby continuously converting the Gibbs free energy stored in the salinity gradient into usable electrical energy [10–12]. In such systems, membrane ion selectivity and conductivity directly govern the open-circuit voltage (Voc) and short-circuit current (I_sc_), and thus determine energy conversion efficiency and power density [11, 12, 17–19]. Consequently, optimizing key membrane properties ion selectivity, ionic conductivity, structural integrity, fouling resistance, and mechanical robustness is essential to improving system performance and enabling long-term operation [10, 14, 18].

**Fig. 1:**
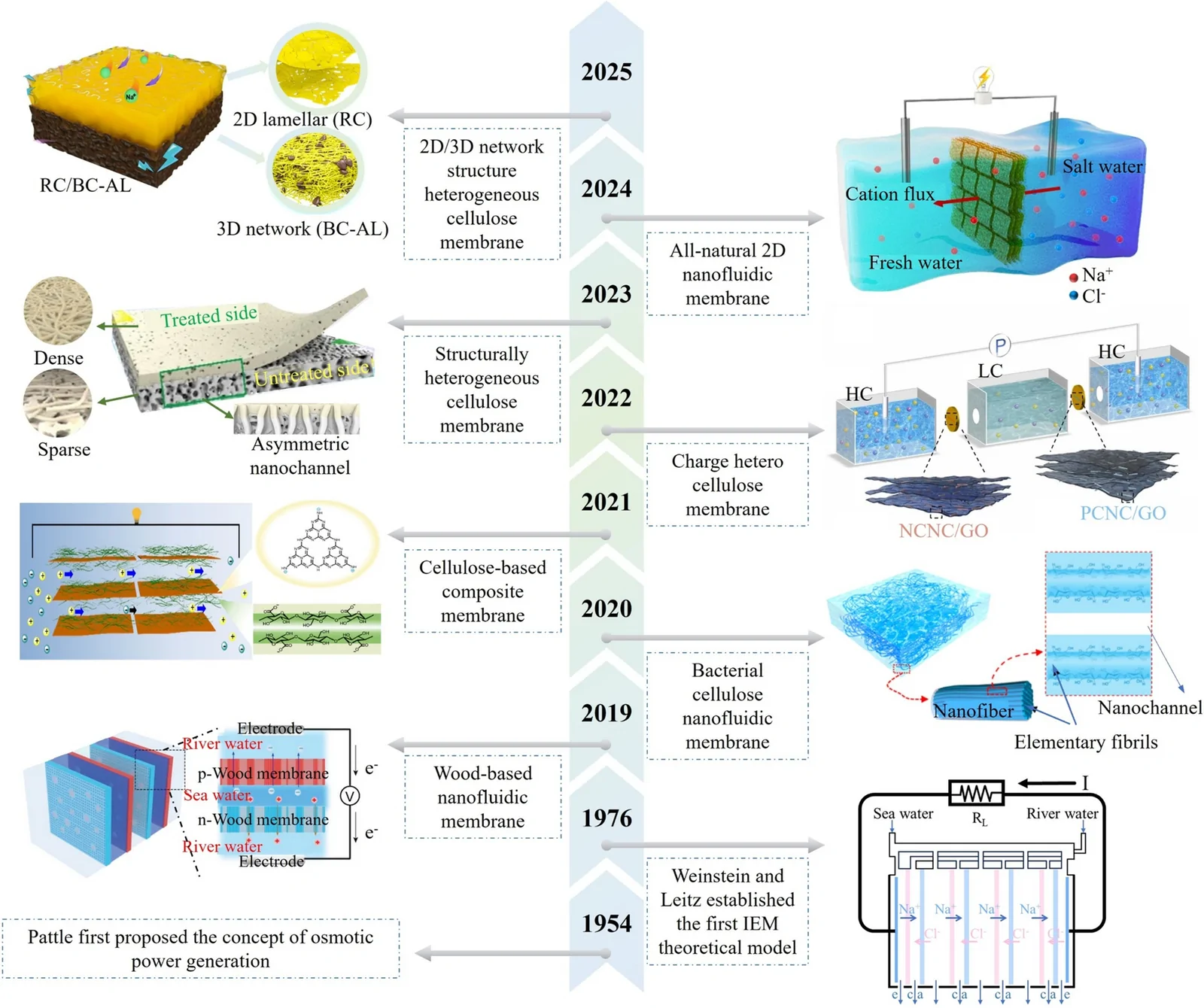
Historical development of osmotic energy conversion, especially based on cellulose nanofluidic membrane [21, 27–34]. Copyright © 1976 reprinted by permission of The American Association for the Advancement of Science. Copyright © 2019 reprinted by permission of John Wiley and Sons. Copyright © 2020 reprinted by permission of Elsevier. Copyright © 2021 reprinted by permission of Elsevier. Copyright © 2022 reprinted by permission of Elsevier. Copyright © 2023 reprinted by permission of Elsevier. Copyright © 2024reprinted by permission of Springer Nature. Copyright © 2025 reprinted by permission of Elsevier

Cellulose, the most abundant natural polymer on Earth, has recently emerged as a promising platform for next-generation green ion-selective membranes [20–23]. Its intrinsic renewability, hierarchical structure, rich surface chemistry, biodegradability, and excellent film-forming ability make cellulose particularly suited for constructing nanofluidic channels with tailored dimensions and surface charges. Cellulose-based membranes are inherently hydrophilic and ionizable, and their morphologies can be tuned from bulk wood to nanocellulose films, regenerated cellulose (RC) membranes, and bacterial cellulose (BC) networks. These characteristics have stimulated interest in cellulose membranes for salinity-gradient power generation, seawater desalination, and related clean-energy technologies (Fig. 1) [24–26].

In this review, we provide a systematic overview of cellulose-based nanofluidic membranes for salinity-gradient energy harvesting, with a particular emphasis on how cellulose’s hierarchical structures and accessible chemistry can be leveraged to program ion transport from the nanoscale to the device level. Different from prior reviews that mainly focus on 2D laminates, COF/MOF nanochannels, or commercial ion-exchange membranes, this review centers on cellulose as a sustainable and structurally programmable platform and offers three differentiated contributions: (1) a cellulose-specific taxonomy spanning wood, nanocellulose, regenerated cellulose, and bacterial cellulose, highlighting their processability-performance trade-offs, (2) design rules that connect surface charge, pore size, porosity, thickness, and heterostructures to selectivity, resistance, and concentration polarization, (3) an engineering-oriented discussion on anti-swelling stabilization, large-area fabrication and corresponding cost, and RED stack integration. We aim to provide actionable guidelines for translating cellulose-enabled nanofluidic from lab-scale demonstrations toward practical blue-energy systems.

## Cellulose Materials in Osmotic Energy Conversion

As the most abundant, natural polymer on Earth, cellulose possesses several desirable characteristics, including renewability, biodegradability, excellent biocompatibility, and low production cost, making it a green and sustainable resource for the development of advanced functional membranes toward ion-selective transport in energy conversion system [35, 36]. The extensive hydrogen bonding within cellulose chains leads to the formation of multi-scaled structures [37], as well as the hierarchical architecture with tunable morphology and dimensions. Such hierarchical structures are advantageous for tailoring nanochannels in membranes, which is critical for achieving selective ion transport for osmotic energy conversion. This unique structural versatility imparts cellulose with excellent processability. In addition, cellulose is rich in hydroxyl groups (-OH), exhibits unique chemical reactivity and potential ionizable groups, rendering it a promising candidate for high-performance osmotic energy conversion (Fig. 1 and Table 1).

**Table 1:** Power density of different types of cellulose

Materials	Concentration gradient	Gross power density (W m^−2^)	Device voltage (V)	Stability (days)	Refs
Wood membrane					
Ionized wood	60-fold NaCl	5.14 × 10^−^^3^	9.8 in 100 pairs	N/R^a)^	[21]
Hydrogel wood	1000-fold KCl	5.6 × 10^−^^4^	N/R	N/R	[38]
Ion-selective wood membranes	50-fold KCl	0.66	N/R	N/R	[39]
PAAS^b)^ hydrogel wood membrane	50-fold NaCl	8.5	1.5 in 7 pairs	N/R	[40]
Nanocellulose membrane					
Nanocellulose/MXene	1000-fold KCl	1.32	N/R	15	[41]
Nanocellulose/g-C_3_N_4_	50-fold KCl	0.15	N/R	30	[30]
Nanocellulose/WS_2_	50-fold NaCl	1.99	1.3 in 30 pairs	N/R	[42]
Nanocellulose/PSS^c)^	50-fold KCl	1.75	N/R	N/R	[43]
Nanocellulose/GO	50-fold NaCl	7.67	N/R	N/R	[44]
T-^d)^ nanocellulose/SPC^e)^ heterogenous membrane	500-fold NaCl	1.47	1.68 in 20 pairs	25	[45]
Nanocellulose/sulfonated polysulfone	50-fold NaCl	8.3	N/R	N/R	[46]
Nanocellulose/BTCA^f)^ cross-linking	50-fold NaCl	8.87 × 10^−^^3^	N/R	N/R	[47]
Nanocellulose/MOF	50-fold KCl	1.87	1.5 in 15 pairs	12	[48]
Regenerated cellulose membrane					
N-^g)^ RC/P-^h)^ RC	50-fold KCl	N-^g)^ RC: 2.27 P-^h)^ RC: 1.28	1.32 in 30 pairs	100	[25]
RCNF^i)^	50-fold NaCl	2.57	3.5 in 24 pairs	43	[49]
RC/CNT^j)^	50-fold NaCl	5.28	2.06 in 20 pairs	50	[50]
RC/PLL^k)^/PET^l)^	5000-fold NaCl	4.9	N/R	N/R	[51]
RC/PVP^M)^	50-fold NaCl	1.33	N/R	14	[52]
Bacterial cellulose membrane					
N-^g)^ BC/P^h)^-BC (chemical modification)	50-fold NaCl	0.23	2.34 in 18 pairs	15	[29]
N-^g)^ BC/P^h)^-BC (in situ culture)	50-fold KCl	N-^g)^ BC: 2.25 P-^h)^ BC: 0.42	2.53 in 15 pairs	15	[53]
N-^g)^ BC/MXene	50-fold KCl	5.3	N/R	N/R	[54]
T-^d)^ BC/GO	50-fold KCl	0.7	3.6 in 24 pairs	15	[55]
BC/MXene Janus membrane	50-fold NaCl	0.91	N/R	N/R	[56]
C-^N)^ BC/GO	500-fold KCl	7.49	3.6 in 28 pairs	20	[57]

Note: ^a)^ N/R: Not reflected in the text; ^b)^ PAAS: sodium polyacrylate; ^c)^ PSS: sodium polystyrene sulfonate; ^d)^ T-: TEMPO-oxidized; ^e)^ SPC: sulfonated polysulfone; ^f)^ BTCA: 1,2,3,4-butanetetracarboxylic acid; ^g)^ N-: indicates negatively charged; ^h)^ P-: indicates positively charged; ^i)^ RCNF: regenerated cellulose-based nanofluidic fibers; ^j)^ CNT: carbon nanotubes; ^k)^ PLL: poly-L-lysine; ^l)^ PET: polyethylene terephthalate; ^m)^ PVP: polyvinyl pyrrolidone; ^n)^ C-:carboxymethyl cellulose sodium salt

### Wood-Based Nanofluidic Membrane

**Fig. 2:**
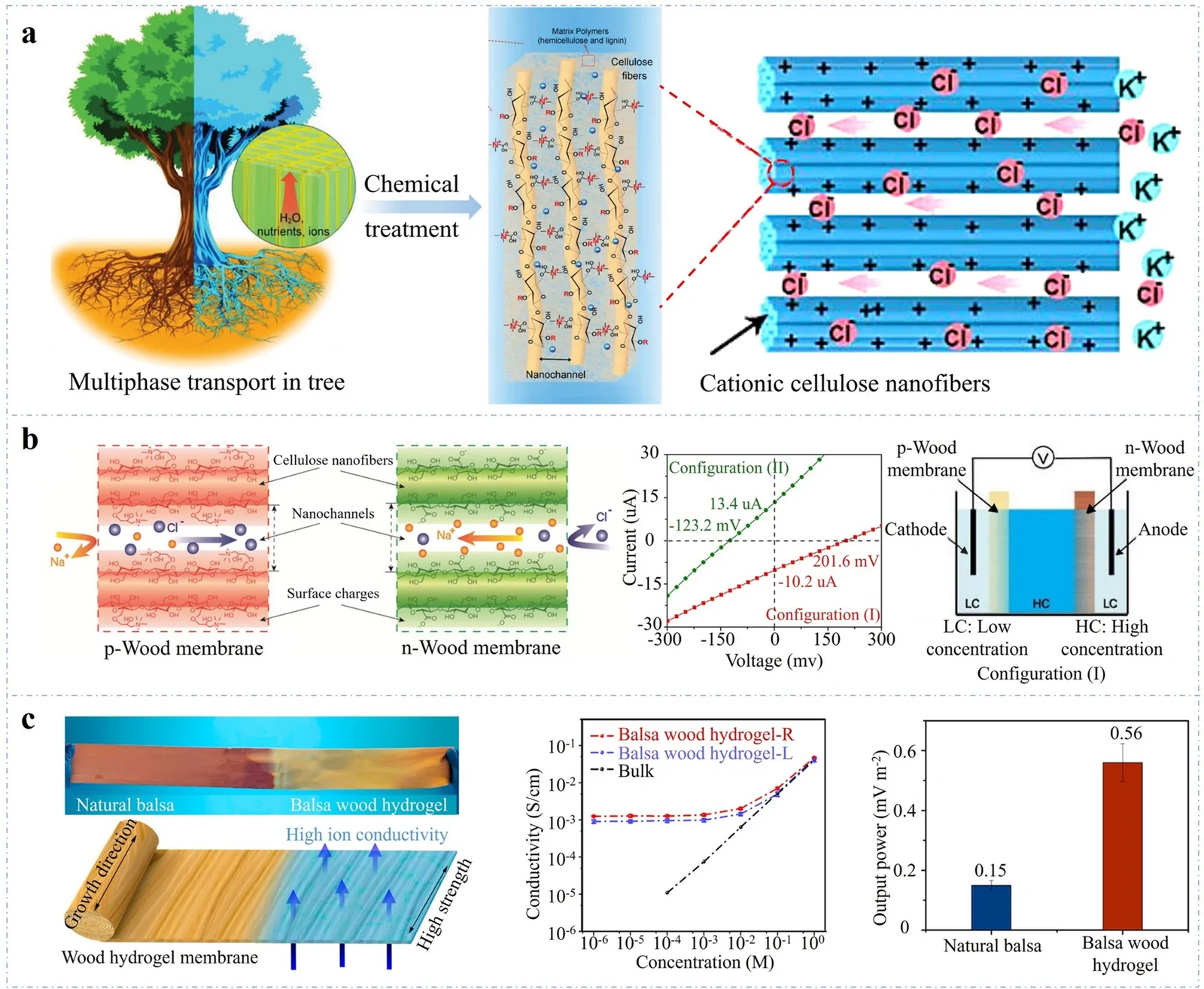
Applications of wood in osmotic energy conversion. **a** Multiphase transport (e.g., water, ions, and nutrients) in active tree [61, 62]. Copyright © 2019 reprinted by permission of John Wiley and Sons. **b** Selective transport of anions and cations in p-wood and n-wood membranes [21]. Copyright © 2019 reprinted by permission of John Wiley and Sons. **c** Fabrication and performance of wood hydrogel membrane [38]. Copyright © 2021 reprinted by permission of American Chemical Society

Wood, as a naturally abundant and renewable material, offers advantages such as low cost, high mechanical strength, and intrinsic hydrophilicity [58–60]. Its microstructure is composed of numerous hollow cells interconnected to form a continuous lumen network, which facilitates the transport of water and nutrients from roots to the canopy (Fig. 2a) [61–64]. The cell walls are primarily composed of cellulose, hemicellulose, and lignin. Through selective chemical treatments, such as partial delignification or hemicellulose removal, the spacing between cellulose nanofibrils becomes more pronounced, forming interconnected nanofluidic channels suitable for salinity gradient energy harvesting [65–68]. Wu et al. [21] exploited the aligned nanofibrillar channels in natural wood and modified the cellulose hydroxyl groups in situ to introduce quaternary ammonium and carboxyl groups, thereby imparting positive (p-wood) or negative (n-wood) charges to the nanochannels. These charged channels served as selective ion-transport pathways that enabled efficient directional ion migration and osmotic energy conversion (Fig. 2b). Under a 60-fold NaCl gradient (0.01/0.6 M, artificial river/seawater), a device assembled from paired ionic wood membranes delivered a power density of 5.14 × 10^−3^ W m^−2^. When 100 pairs were connected in series, an output voltage of 9.8 V was achieved.

Although natural wood membrane demonstrates inherently selective ion transport, their relatively large pore sizes (much greater than the Debye length) result in low ion selectivity due to the mismatch between the pore size and the Debye length, which poses considerable challenges for practical implementation. In particular, poor structural stability in aqueous environments and insufficient surface charge density represent performance bottlenecks. To address these issues, Chen et al. [38] developed a balsa wood hydrogel membrane by in situ polymerization of polyvinyl alcohol (PVA) and acrylic acid (AA) within the nanochannels formed by aligned cellulose nanofibrils (Fig. 2c). The resulting polymer hydrogel filled the macropores of the wood membrane, increasing the number of nanosized pores, which serve as ion transport pathways, and the surface charge density. More importantly, the tensile strength of the hydrogel-modified wood membrane reached 52.7 MPa, three times that of the native balsa wood membrane. The increased density of nanochannels and surface charges enabled an ionic conductivity of 1.29 mS cm^−1^ (Fig. 2c). Under a 1000-fold salinity gradient, the system achieved a power density of 5.6 × 10^−4^ and 2.7 × 10^−3^ W m^−2^ at the dosage of AA of 25 and 50 wt%, respectively.

In summary, wood-derived nanofluidic membranes leverage naturally aligned channels and robust frameworks, but their broad pore-size distributions, limited surface charge, and structural deformation in water still pose challenges. Rational functionalization and hybridization are therefore essential to push wood membranes toward high-performance osmotic energy harvesting.

### Nanocellulose Nanofluidic Membrane

**Fig. 3:**
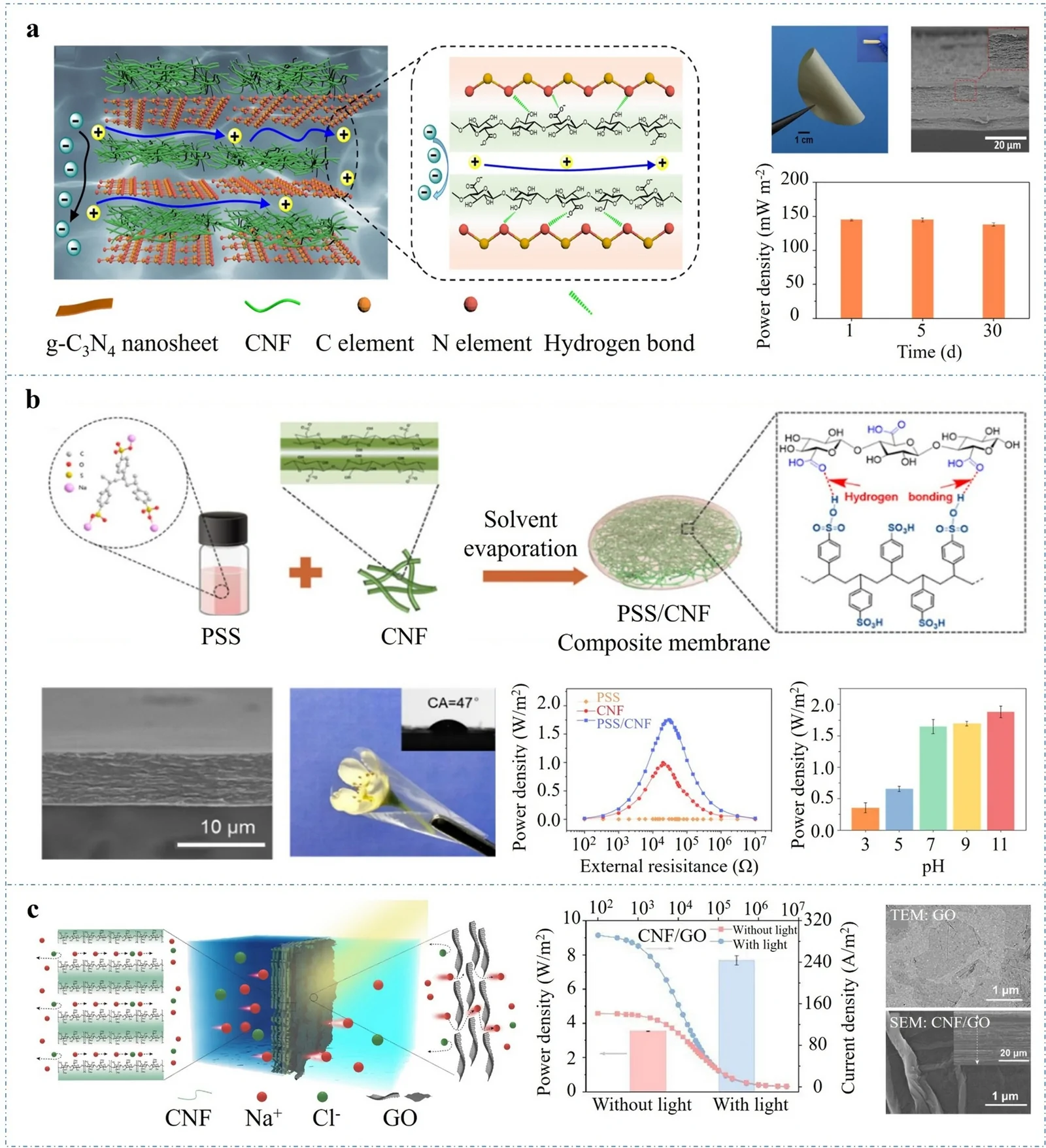
Application of nanocellulose in osmotic energy conversion. **a** Fabrication and ion transport performance of nanocellulose/g-C_3_N_4_ composite nanofluidic membrane [30]. Copyright © 2022 reprinted by permission of Elsevier. **b** Fabrication and ion transport performance of nanocellulose/PSS composite nanofluidic membrane [43]. Copyright © 2024 reprinted by permission of American Chemical Society. **c** Fabrication and ion transport performance of nanocellulose/GO composite membrane [44]. Copyright © 2022 reprinted by permission of American Chemical Society

Compared with bulk wood, nanocellulose such as cellulose nanofibers (CNFs) and cellulose nanocrystals (CNCs) offers higher structural tunability and dense networks of surface functional groups (e.g., –COOH, –OH) [69–72]. Nanocellulose exhibits excellent film-forming ability, enabling flexible fabrication of membranes with controllable thickness, porosity, and surface chemistry. Furthermore, nanocellulose readily forms composites with two-dimensional (2D) materials and charged polymers, providing powerful knobs for regulating ion-transport channels [6, 73]. For instance, Gao et al. [30] systematically investigated the effect of g-C_3_N_4_ on the ion transport in the composite nanocellulose/g-C_3_N_4_ nanofluidic membrane, and pointed out that g-C_3_N_4_ facilitated the ion diffusion through the nanochannels via enhanced electrostatic interactions and Donnan exclusion effects. Under simulated seawater/river water conditions, the power density reached 0.15 W m^−2^ (Fig. 3a). Beyond inorganic 2D materials, charged polymers can also be integrated. Li et al. [43] designed a nanofluidic membrane composed of sulfonic acid-rich sodium polystyrene sulfonate and nanocellulose (Fig. 3b). By optimizing the content of sodium polystyrene sulfonate, the nanofluidic membrane exhibited a power density of 1.75 W m^−2^ under a 50-fold KCl concentration gradient (0.01/0.5 M), significantly outperforming pure nanocellulose membrane (Fig. 3b). In addition, researchers have further introduced sub-nanometer channel “enhancers” into nanocellulose matrices to overcome the conventional permeability-selectivity trade-off in nanocellulose membranes. For example, MOF nanocrystals in-situ grown on the surface of sulfated CNCs can provide additional nano-/sub-nano ion transport pathways, thereby synergistically improving both the ionic conductivity and monovalent ion selectivity of the membrane while preserving the sustainability advantages of the biomass-derived framework [74]. On the other hand, a nacre-mimetic self-assembled cellulose composite membrane is constructed by incorporating flexible PVA and graphene oxide (GO) into CNC system, which markedly enhances the wet-state mechanical strength and dimensional stability of the CNC membrane, and effectively alleviate the tendency of highly charged nanocellulose networks to soften and lose structural stability under hydrated conditions [75].

Noted that photothermal strategies have also been employed to improve the energy conversion performance of nanocellulose membrane, where the system temperature increased spontaneously to enhance the transport speed of ions. Luo et al. [44] fabricated a nanocellulose/low-dimensional carbon (LDCM) composite membrane, in which nanocellulose served as the ion-selective layer, and LDCM enabled photothermal energy conversion to raise the system temperature. Under lateral light irradiation, the engineered composite membrane raised the system temperature to 53 °C and correspondingly demonstrated a high power density of 7.67 W m^−2^ with a NaCl concentration gradient of 0.01/0.5 M, substantially higher than that in dark condition (3.55 W m^−2^ at 25 °C in Fig. 3c).

Despite the promising performance of nanocellulose nanofluidic membrane in the read tunability of structural and chemical distinction, and further improved energy conversion efficiency, several critical challenges remain: (1) Incompatibility between organic nanocellulose and inorganic 2D material can result in poor interfacial strength and reduced membrane stability for long-term energy conversion; (2) Nanocellulose tend to aggregate during the manufacturing process of nanofluidic membrane, compromising membrane uniformity and performance consistency; (3) Based on the traditional vacuum filtration, the scalability and processability of nanocellulose composite membrane is challenging.

### Regenerated Cellulose Nanofluidic Membrane

In lignocellulosic plants, cellulose molecules first assemble into nanoscale cellulose fibrils and then into a large-scale wood block. Hence, cellulose molecules offer obvious advantages in chemical and structural functionalization, enabling the construction of tailored high-performance membrane materials. Based on the dissolution-regeneration method, the RC membrane is fabricated via a bottom-up assembly of cellulose molecules. Its fabrication is highly controllable, allowing precise tuning of membrane structure, pore distribution, and surface charge properties, making it suitable for various osmotic energy conversion applications [76–78].

**Fig. 4:**
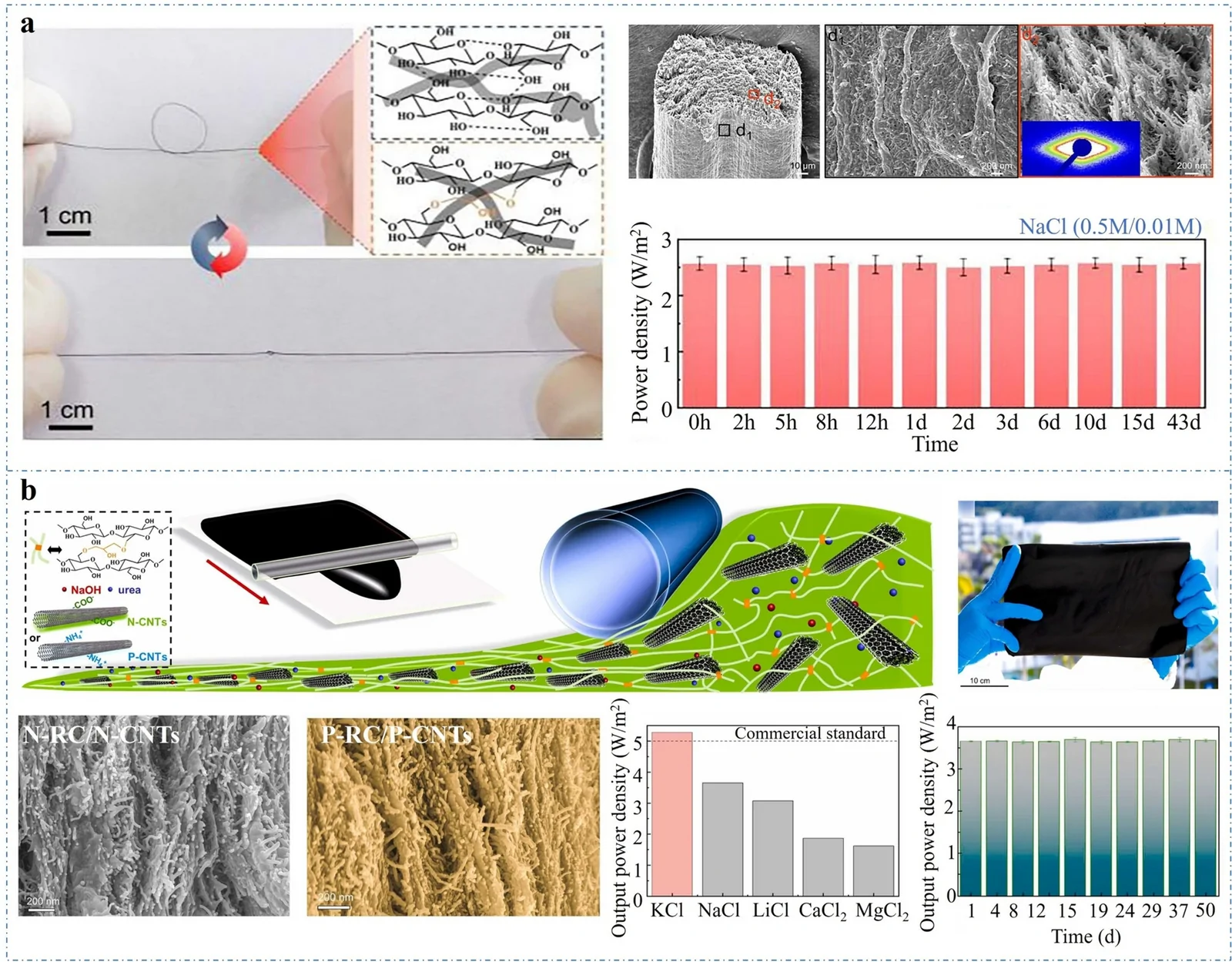
Applications of regenerated cellulose in osmotic energy conversion. **a** Regenerated cellulose-based fiber membrane [49]. Copyright © 2022 reprinted by permission of Elsevier. **b** RC/CNTs nanohybrid membrane [50]. Copyright © 2023 reprinted by permission of Elsevier

During the manufacturing process of RC membrane, by tailoring solvent systems, coagulation bath parameters, and additive composition, fiber-like or sheet-like membrane with oriented alignment, as well as rich nanochannels and surface charges, can be achieved. Zhou et al. [49] proposed the regenerated cellulose-based nanofluidic fibers (RCNF) by integrating cellulose dissolution, alignment, regeneration, and densification processes (Fig. 4a), Due to the synergistic orientation and spatial confinement effect of the cross-linked cellulose network, RCNF achieved high power density (2.57 W m^−2^ over 43 days) in an artificial river water-seawater system, far exceeding the performance of some commercial ion-exchange membrane. In addition, Zhou et al. [50] developed a simple solution-casting method to prepare a directionally RC/CNT nanofluidic membrane (Fig. 4b). Under a 50-fold salinity gradient, the membrane achieved a power density of 5.28 W m^−2^, surpassing the benchmark value for commercial PRO membrane (5 W m^−2^) [79] with stable performance over a 50-day period.

Cellulose molecules expose abundant hydroxyl groups, which form the sufficient inter- and intra-hydrogen bonds, and endow the high strength to the RC membrane. Such mechanical characterizations would ensure the structural stability of the RC membrane in a water environment (due to its hydrophilic nature, cellulose materials commonly deform in aqueous systems) for long-term operation. Shi et al. [25] through self-assembly engineering of cellulose molecules, constructed the high-charged yet strong RC membrane. Under a 50-fold KCl gradient, the membrane exhibited a high power density of 2.27 W m^−2^ and an ultrahigh voltage of 1.32 V. Moreover, the self-assembly RC membrane demonstrated high mechanical strength (4.2 MPa for negative CMA membrane) and long-term structural and dimensional integrity in saline environments, maintaining stable power output for over 100 days.

Despite the notable advantages of RC in structural and chemical tunability, as well as stability, several challenges remain in their application for osmotic energy conversion: (1) During the manufacturing RC membrane (dissolution and regeneration), some bubbles remains exist in the viscous cellulose solution, which would lead to the micro-sized pores rather than the nanochannels, and finally decreases the ion transport capability; (2) The dissolution and regeneration process is typically time-and energy-consuming, and result in the high cost.

### Bacterial Cellulose Nanofluidic Membrane

Bacterial cellulose (BC) is primarily synthesized by microorganisms such as Acetobacter species under suitable conditions. Its main component is highly pure cellulose, with no lignin or other impurities, thereby avoiding the complex steps of lignin removal that occur in natural lignocellulosic materials. Moreover, the unique biosynthetic process of BC results in a three-dimensionally interconnected nanoscale network structure, which enhances membrane porosity and mechanical strength, offering improved stability during osmotic energy conversion [80, 81].

**Fig. 5:**
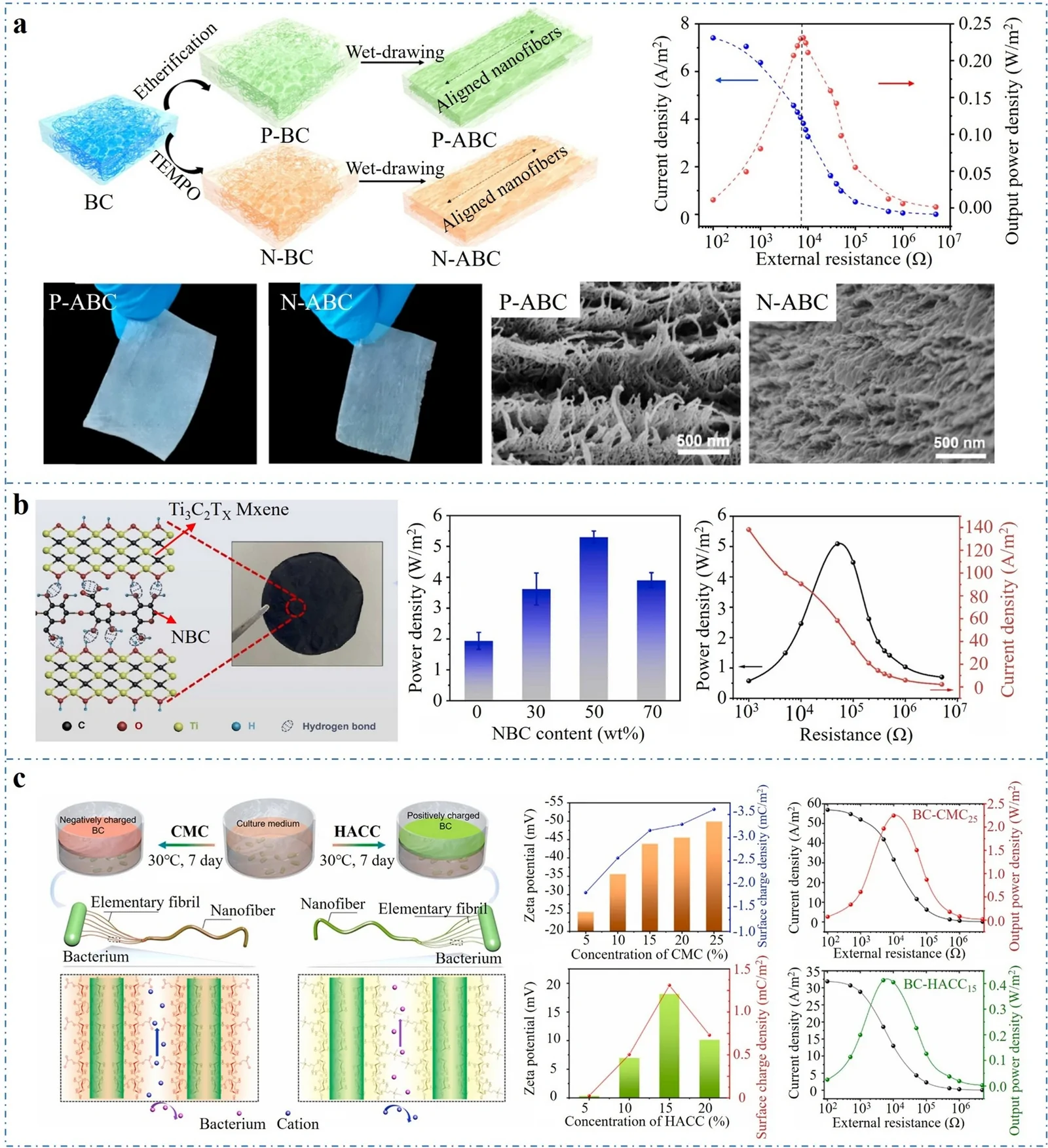
Applications of BC in osmotic energy conversion. **a** Ionized BC membranes [29]. Copyright © 2020 reprinted by permission of Elsevier. **b** NBC/MXene composite membrane [54]. Copyright © 2022 reprinted by permission of Elsevier. **c** Charged BC membrane fabricated via in situ microbial fermentation [53]. Copyright © 2021 reprinted by permission of Elsevier

Recent studies have demonstrated that both chemical surface modifications (e.g., TEMPO-mediated oxidation, quaternization, or phosphorylation) and structural composites can effectively enhance ion selectivity, thereby improving the osmotic-energy conversion performance of BC membrane. For example, Wu et al. [29] converted pristine BC into positively charged (P-BC) and negatively charged (N-BC) membranes via quaternization and TEMPO-mediated oxidation treatments (Fig. 5a), resulting in a substantial increase in surface charge density, 5.25-fold and 12.5-fold higher than that of native BC, respectively, thereby improving the ionic conductivity of the chemical-modified BC membrane. Moreover, the device assembled from this charged BC membrane achieved a power density of 0.23 W m^−2^ under simulated river water/seawater conditions (0.01/0.5 M) and, by connecting 18 units in series, a maximum output voltage of 2.34 V. Similarly, Wang et al. [54] fabricated composite membrane by integrating N-BC with MXene nanosheets, in which the embedding of one-dimensional BC nanofibers into the two-dimensional MXene nanosheet framework generated a space-charge-enhancement effect, synergistically improving both ion selectivity and ion flux. The resulting N-BC/MXene composite membrane exhibited high energy conversion efficiency and good biocompatibility, achieving a power density of 5.3 W m^−2^ under a 50-fold salinity gradient (0.01/0.5 M) (Fig. 5b).

Moreover, the structural and chemical distinctions of BC can be controlled by optimizing its biosynthesis conditions, such as medium pH, carbon-source composition, and cultivation temperature, which would affect the water flux and ion selectivity of BC membrane. Wu et al. [53] employed the culture medium with sodium carboxymethyl cellulose (CMC) and quaternized chitosan (HACC), and further easily prepared negatively charged N-BC membrane (surface charge density of − 3.16 mC m^−2^) and positively charged P-BC membrane (surface charge density of 1.31 mC m^−2^), which exhibited power output of 2.25 and 0.42 W m^−2^ (Fig. 5c), respectively, under the artificial seawater/river water concentration gradient (0.01/0.5 M). Furthermore, serially connecting 15 units of N-BC and P-BC membranes yielded an output voltage of 2.53 V, sufficient to directly power electronic devices.

These findings indicate that through structural design and chemical modification, the ion selectivity and corresponding energy conversion efficiency of BC membrane can be significantly improved, providing new insights for the development of high-performance, environmentally friendly membrane for salinity gradient energy harvesting. However, several limitations hinder the practical application of BC membrane: (1) The microbial biosynthesis of BC is time-consuming, with limited yield, making it difficult to meet the demands of large-scale production. (2) Although the nanofiber network enhances rigidity, BC membrane is still prone to cracking or fracturing under dry or high-humidity conditions. (3) Under non-ideal growth conditions, the variability in pore size and distribution may compromise the membrane reproducibility and long-term stability.

## Design Principle of Nanofluidic Membrane of Cellulose

The efficiency and stability of salinity-gradient energy conversion depend critically on membrane structure and properties. Ideal membranes require high ion selectivity, low transport resistance, strong mechanical robustness, excellent chemical stability, and antifouling performance [82–85]. Achieving this combination hinges on precise control of micro- and nanoscale structural and chemical parameters, including surface charge, pore size, porosity, thickness, and heterostructure design. In this section, we summarize how these design levers regulate ion transport and energy conversion in cellulose-based nanofluidic membranes.

### Surface Charge

**Fig. 6:**
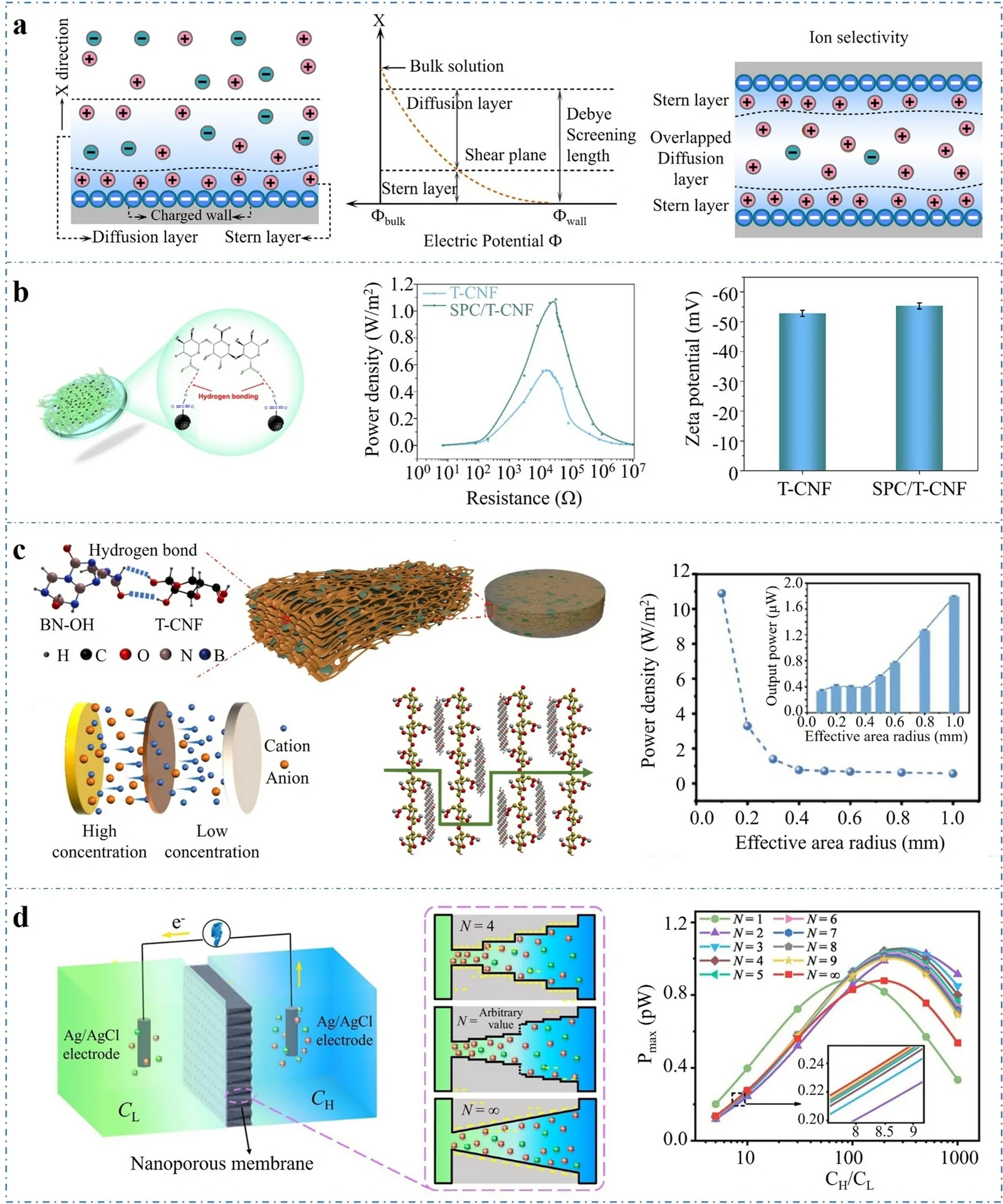
Surface charge regulation. **a** Schematic illustration of the EDL mechanism near negatively charged walls and ion selectivity in surface-charged nanochannels [90]. Copyright © 2019 reprinted by permission of Elsevier. **b** Design and performance of CEMs [45]. Copyright © 2025 reprinted by permission of Elsevier. **c** Effect of surface charge distribution uniformity on membrane performance [92]. Copyright © 2024 reprinted by permission of Elsevier. **d** Effect of surface roughness on membrane performance [99]. Copyright © 2025 reprinted by permission of Elsevier

Surface charge regulation is one of the key strategies for achieving high ion selectivity and efficient energy conversion in cellulose-based membrane [86, 87]. Its primary mechanism involves controlling charged-nanofluidic channels to enable the selective transport of cations and anions in the system. Within the inner walls of charged nanochannels, electrostatic forces repel co-ions (ions with the same charge as the channel) and attract counter-ions (ions with the opposite charge), leading to counter-ion enrichment near the channel walls [24, 88]. Driven by thermal motion and electrochemical potential, a stratified electric double layer (EDL) is formed adjacent to the nanochannel walls, resulting in increased counter-ion concentration and reduced co-ion concentration (Fig. 6a). When the nanochannel size approaches the Debye length, the EDL regions overlap within the channel, causing ion transport to be governed by surface charge. This leads to counter-ion enrichment and pronounced ion selectivity (Fig. 6a) [89, 90].

The cellulose molecules contain abundant hydroxyl groups, which can impart a weakly negative surface charge in aqueous environments. However, due to the limited ionization capacity of hydroxyl groups, cellulose membranes generally exhibit poor ion selectivity in saline solutions. By introducing negatively or positively charged functional groups (e.g., –SO_3_H, –COOH, –NH_2_, and –NR_3_^+^) onto the cellulose chains, a stable electrostatic environment can be established at the membrane surface, thereby significantly influencing its ion selectivity, ion transport rate, and electrical conductivity [91, 92]. According to the polarity of the surface charge, cellulose membranes can be tailored as CEMs or AEMs, enabling selective transport of cations or anions, respectively, and are widely employed in salinity-gradient energy conversion.

Common strategies for developing CEMs include TEMPO-mediated oxidation, sulfonated polydopamine (SDA) modification, and composite fabrication with sulfonated carbon materials (e.g., SPC) [93–95]. He et al. [45] introduced carboxyl groups by TEMPO-oxidized treatment and sulfonic groups by incorporating sulfonated nanoporous carbon into the cellulose membrane, which enabled the high zeta potential to − 54 mV to the cellulose membrane, thereby promoting cation adsorption and co-ion exclusion, resulting in enhanced cation selectivity (0.88) and an improved energy conversion efficiency of 38.3%. Furthermore, the resultant membrane exhibited excellent ionic conductivity (0.8 S cm^−1^) under low KCl concentrations (≤ 0.01 M), achieving a balance between high ion selectivity and fast ion transport. Under simulated seawater/river water conditions, the cellulose membrane delivered a power density of 1.08 W m^−2^, and maintained a stable performance over 25 days of continuous operation (Fig. 6b). Notably, the sulfonic acid groups, as strongly acidic ionizable moieties, are able to fully dissociate in aqueous environments, thereby substantially increasing the fixed negative charge density at the membrane surface. Such enhancement in surface charge not only reinforces the electric double-layer effect but also facilitates the preferential transport of cations, consequently reducing ion migration resistance and improving the overall efficiency of energy conversion. For instance, Shi et al. [24] incorporated sulfonic acid groups into a cellulose-based membrane, leading to a remarkable increase in surface zeta potential from − 23 to − 44 mV, which significantly enhanced ion transport capability. Owing to this charge-regulation effect, the PCC membrane achieved a power density of 5.3 W m^−2^, under a 50-fold NaCl concentration gradient, substantially higher than that of the pristine oxidized cellulose (OC) membrane (0.94 W m^−2^). Thereby fully verifying the key role of sulfonic acid groups in enhancing osmotic energy conversion performance.

In its natural state, the surface of cellulose is typically decorated with hydroxyl groups or partially carboxyl groups, rendering it overall negatively charged and resulting in a lack of selectivity for anion transport. Therefore, to achieve anion selectivity (i.e., preferential transport of Cl^−^, NO_3_^−^, etc.), positively charged functional groups are typically introduced onto cellulose chains to construct AEMs. Commonly, fabrication strategies include epichlorohydrin amination, polyethyleneimine (PEI) crosslinking, and surface polymerization of amine-containing small molecules. These methods markedly increase the surface positive charge density of the cellulose membrane. For instance, Zhang et al. [96] applied quaternary ammonium modification to natural cellulose to prepare a positively charged cellulose membrane (PPC), with its surface zeta potential increased from − 25 to + 14 mV. Benefiting from the abundant cationic functional groups and uniformly distributed nanoscale pores, the PPC membrane achieved a high power density of 2.2 W m^−2^ under a 0.01/0.5 M salinity gradient, which is three times higher than that of the pristine cellulose membrane. Moreover, after continuous immersion in solutions with pH ranging from 3 to 12 for 300 days, the membrane maintained excellent structural integrity and electrochemical stability. Notably, although quaternization partially disrupted the hydrogen-bonding network between cellulose chains, the phase-inversion-induced densified structure enabled the PPC membrane to retain a tensile strength of 89.8 MPa in 0.2 M electrolyte. This value meets the > 50 MPa criterion for practical stack assembly and ensures mechanical reliability under high-pressure differentials and long-term operational conditions.

Beyond charge polarity, other factors such as the surface charge distribution, surface roughness, and nanochannels architecture also play critical roles in governing ion transport behavior and energy conversion efficiency [92, 97, 98]. Localized heterogeneity in surface charge distribution can induce spatial variations in the surface electric field, thereby altering ion enrichment locations and migration pathways (Fig. 6c) [92]; Similarly, higher surface roughness or the presence of porous structures can markedly increase the electroactive surface area, facilitating the transmembrane flux of hydrated ions (Fig. 6d) [99]. Moreover, when the surface charge density becomes excessively high, strong electrostatic interactions and charge-screening effects may alter ion selectivity and diffusion behavior [100, 101].

In summary, molecular design and precise regulation of surface charge provide a key approach to enhance ion selectivity and energy conversion efficiency in cellulose-based membranes. Although current cellulose membranes have demonstrated high power output and stability, future advances in the precise control of charge density and spatial distribution, and the incorporation of environmentally responsive functional modifications are expected to enable high-performance, durable, and sustainable salinity-gradient energy conversion systems.

### Pore Diameter

**Fig. 7:**
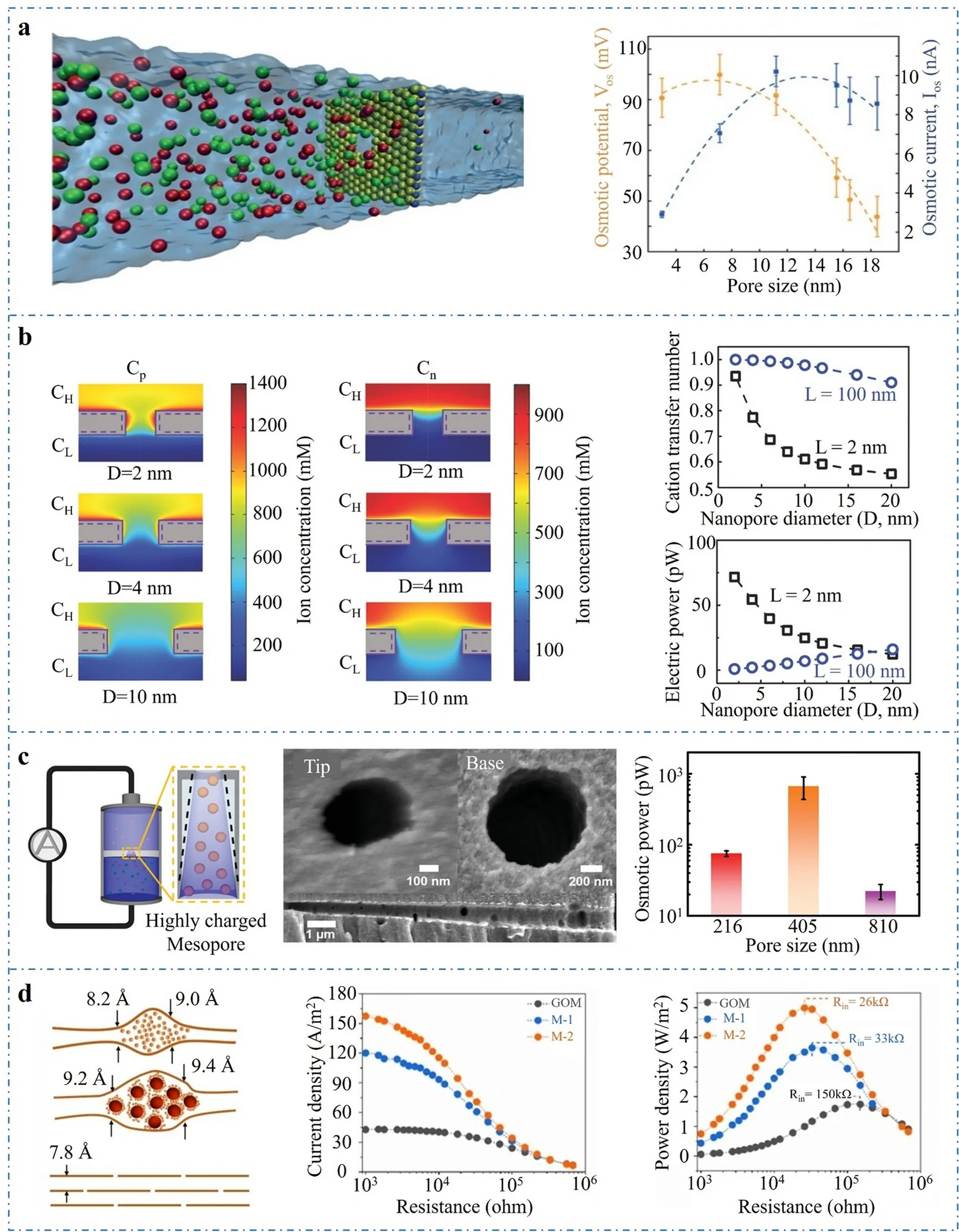
Effects of pore diameter on osmotic energy conversion. **a** Trade-off relationship between pore size, output voltage, and osmotic current [107]. Copyright © 2016 reprinted by permission of Springer Nature. **b** Disruption of membrane charge selectivity caused by anion back diffusion induced by increased pore size [103]. Copyright © 2018 reprinted by permission of John Wiley and Sons. **c** Efficient ion transport enabled by the synergistic effect of high surface charge and asymmetric geometry [110]. Copyright © 2020 reprinted by permission of John Wiley and Sons. **d** Achieving high cation selectivity in non-subnanometer channels via the “channel expansion-enhanced space charge” strategy [111]. Copyright © 2022 reprinted by permission of John Wiley and Sons

Pore diameter is a key parameter influencing the energy conversion performance of ion-selective membranes, as it directly affects ion selectivity, ion flux, and ultimately the power density [102–105]. During the energy conversion, pore size significantly affects the output voltage (E_m_) and osmotic current (I_osm_). Specifically, decreasing the pore diameter increases the overlap of the EDL, thereby enhancing the membrane’s ion selectivity, and increasing the permeation voltage [106, 107]. Although a small-pore-size structure can significantly enhance E_m_ [106, 108], further reduction in pore diameter would lead to an obvious increase in channel resistance and reduction in ion flux, ultimately decreases I_osm_. Such a decline of I_osm_ offsets the power gain achieved through the increased E_m_ (Fig. 7a) [107]. Therefore, membrane performance is constrained by the trade-off between voltage and current output, leading to a nonmonotonic trend in osmotic energy conversion efficiency. An optimal pore diameter therefore exists, corresponding to a balance between EDL overlap and ionic conductance, at which maximum power output is achieved [13, 109].

Feng et al. [107] studied the nanochannels of single-layer molybdenum disulfide (MoS_2_), and systematically revealed how pore size regulates energy conversion performance. By varying the channel diameter, they quantitatively characterized the trade-off between output voltage and osmotic current, determining an optimal diameter of ≈10.2 nm. At this optimal diameter, the EDL overlap and ionic flux are optimally coupled, yielding a peak osmotic power density of 10^6^ W m^−2^ (Fig. 7a). Notably, as the pore diameter decreases further, the EDL becomes fully overlapping within the pore, and the ion selectivity coefficient Σ(S) approaches 1, exhibiting ideal cation selectivity. However, under such strong confinement, the total current decreases markedly because the effective conductive region is restricted to the EDL, where charge separation and concentration gradients dominate the ion transport. As the channel cross-sectional area sharply diminishes, the number of mobile ions becomes limited, exhibiting a “depletion” effect, while the geometric resistance increases markedly, leading to a substantial reduction in ion flux and total current, and ultimately causing the power output to transition from rising to declining. This “initial enhancement followed by limitation” trend is not confined to MoS_2_ channels and has also been observed in bullet-shaped etched nanochannels of polyethylene terephthalate (PET) membrane [106], further supporting its universality.

Furthermore, Cao et al. [103] systematically demonstrated that even a slight increase in pore size will cause anions to enter from the high-concentration side, thereby significantly disrupting the membrane's charge selectivity (Fig. 7b). To address the issue of insufficient selectivity caused by large pore diameters, researchers sought to enhance ion selectivity by modifying the surface charge. For example, Gao et al. [110] introduced a high density of negative charges (−160 mC m^−2^ at pH = 11) on the surfaces of 405 nm conical PET mesopores. By combining the asymmetric confinement of the conical pores with the high mesoscale surface charge, the mesopores maintained strong rectification and cation selectivity (t_+_ = 0.832) without requiring electrical double-layer overlap. At the same time, the larger pore size significantly reduced channel resistance and increased ion flux, boosting the osmotic conductance to 0.284 μS, the current to 27.5 nA, and achieving an ultrahigh power output of 667 pW, more than double that of existing single-nanopore devices. This work demonstrates that the synergistic design of high surface charge and asymmetric geometry can construct efficient ion transport channels at submicron pore sizes, providing new strategies for designing mesoscale osmotic energy conversion devices (Fig. 7c). Similarly, Qian et al. [111] introduced spindle-shaped nanochannels (2–8 nm in height) between graphene oxide layers and prefilled them with polyanion electrolytes (PSS). Through a “channel expansion and enhanced spatial charge” strategy, high cation selectivity (t_+_ > 0.9) and low transport resistance were maintained without compressing the channels to sub-nanometer scales. Under a 50-fold NaCl gradient, a practical power density of 4.94 W m^−2^ was achieved, approximately 7 times higher than that of the original GOM (0.7 W m^−2^), and further increased to 34.1 W m^−2^ when using HCl as the electrolyte (Fig. 7d). Similarly, studies have shown that by introducing high surface charge density on the large-scale pore walls, nonlinear ion current rectification (ICR) behavior can be induced, compensating for its inherent selectivity deficiency [112–115], broadening the feasible range of pore size design.

### Porosity

In addition to pore size, the porosity also significantly affects osmotic energy conversion efficiency of the membrane. Porosity determines the density of effective transport channels and is an important structural factor influencing ion migration rate and correspondingly output power [116–119]. Moderate increases in membrane porosity enhance overall ion migration rate and output current density, thereby substantially improving power density under large salinity gradients.

**Fig. 8:**
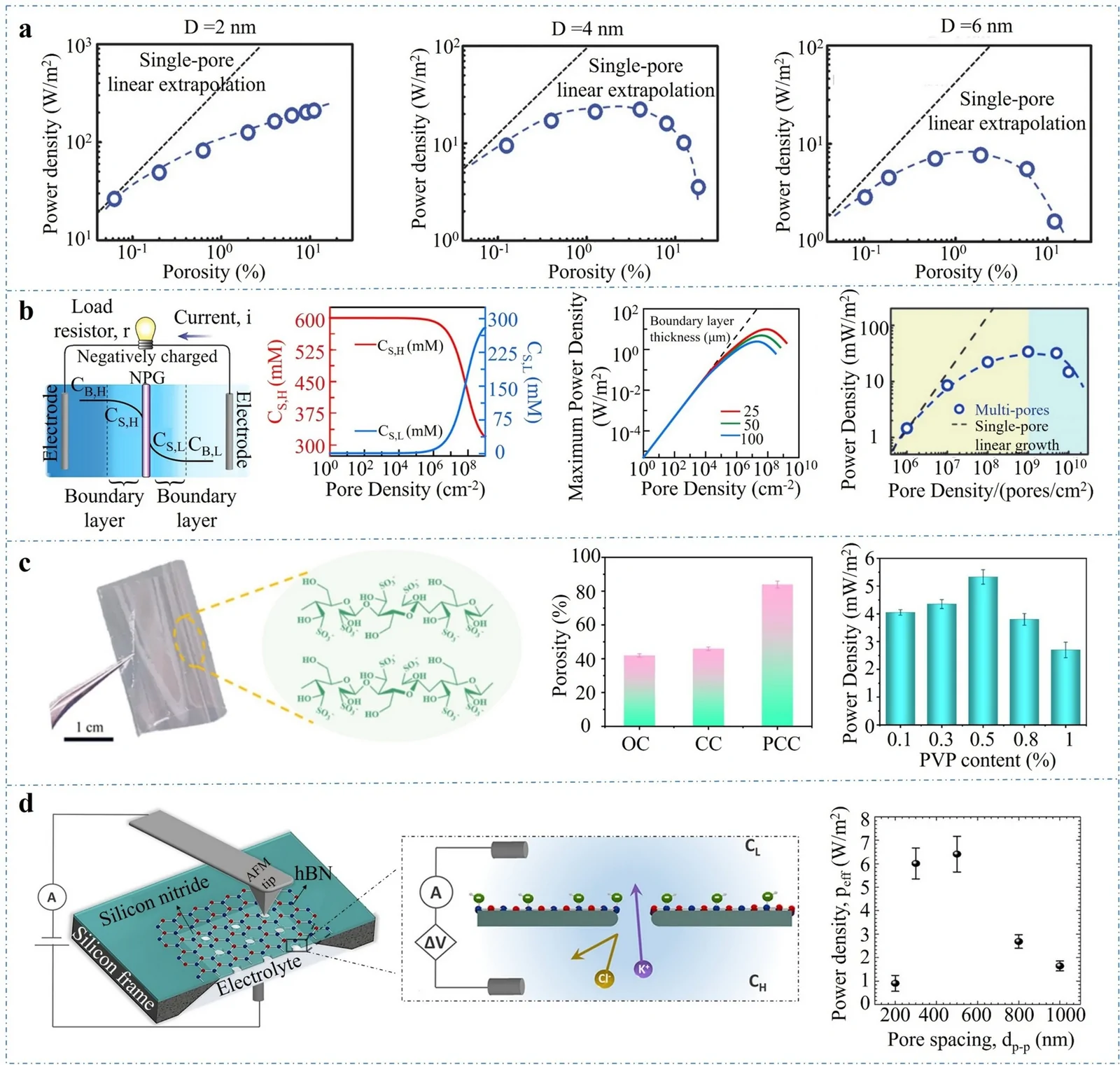
Effects of porosity on osmotic energy conversion. **a** Power density of ultrathin nanoporous membranes with various pore diameters and densities [103]. Copyright © 2018 reprinted by permission of John Wiley and Sons. **b** Anomalous pore density dependence in nanofluidic osmotic energy conversion [13],^125^]. Copyright © 2021 reprinted by permission of American Chemical Society. Copyright © 2018 reprinted by permission of John Wiley and Sons. **c** Porosity and power density of PCC membranes [24]. Copyright © 2024 reprinted by permission of Elsevier. **d** Effect of pore spacing on membrane power density [126]. Copyright © 2021 reprinted by permission of American Chemical Society

At low porosity levels, the total power density of the membrane approximately follows a linear extrapolation based on single-pore devices [103]. However, as the porosity increases, this linear relationship is disrupted [120]. When the porosity is elevated, particularly in membranes with relatively large nanopore sizes (e.g., 4–6 nm), the interactions between adjacent pores are significantly enhanced, leading to strong ion concentration polarization (ICP) effects. Such ICP behavior causes a progressive saturation of power-density growth, with the membrane reaching a maximum at a moderate porosity of approximately 2–4 nm. Beyond this optimal range, further increase in pore-density leads to a decline of power output. In contrast, when the pore size is small (≈2 nm), the performance deterioration induced by high porosity can be partially mitigated, primarily because such confined pores preserve strong EDL overlap and effective ion selectivity (Fig. 8a) [103]. Therefore, under high-porosity conditions, adjusting the surface charge density of the pore exterior can improve overall ion selectivity [121–123]. Notably, the enhancement of the ICP effect not only depletes the local concentration gradient and weakens ion selectivity but also markedly increases the interfacial resistance between the reservoir and the nanochannels, collectively suppressing the overall power output [124]. Moreover, simulation studies indicate that the relationship between power density and porosity is not simply positive; instead, it exhibits a local maximum trend, suggesting the existence of an “optimal porosity” [13, 103, 125]. For instance, Wang et al. [13] simulated that when pore density exceeds 10^6^ pores cm^−2^, the ICP effect severely disturbs the ion concentration distribution at pore entrances, resulting in a decline in unit-pore conductivity (Fig. 8b). Similarly, Su et al. [125] experimentally confirmed a comparable conclusion: when the pore density increased to 1 × 10^9^ pores cm^−2^, the total power output began to decrease (Fig. 8b).

To address the adverse effects of high porosity, researchers have proposed countermeasures in two main directions: pore structure regulation and membrane material system optimization. On the one hand, Shi et al. [24] developed a cellulose membrane with a highly porous and charged structure by combining cellulose sulfonation with the insertion and subsequent removal of PVP nanoparticles during the dissolution-regeneration process. The resulting membrane exhibited a porosity of up to 84%, significantly higher than that of the original cellulose membrane (42%). Notably, under high porosity conditions, the cellulose membrane achieved excellent current density (153.8 A m^−2^) and power density (5.3 W m^−2^) in artificial seawater/river water conditions (Fig. 8c). Laucirica et al. [106] constructed bullet-shaped nanopores via track-etching in PET membrane and demonstrated the feasibility of synergistic regulation of pore size and porosity for enhancing energy conversion efficiency.

On the other hand, Yazda et al. [126] employed a tip-controlled local breakdown (TCLB) method using an atomic force microscope to precisely fabricate nanopore arrays with pore diameters of approximately 6 nm and tunable interpore spacing ranging from 100 to 1000 nm on hBN/SiN hybrid membranes, thereby systematically optimizing the porosity of the membrane material. They found that when the interpore spacing was around 500 nm, the number of pores per unit area and the interpore charge-shielding effect reached an optimal balance, enabling the membrane to achieve the maximum effective power density (15 W m^−2^) while maintaining high ion selectivity. This strategy effectively resolved the issues of charge shielding and concentration polarization associated with high porosity, significantly enhancing osmotic energy conversion efficiency (Fig. 8d).

### Membrane Thickness

The membrane thickness, i.e., the length of the nanochannels, is a crucial structural parameter that influences ion transport pathways, ion selectivity, and osmotic energy conversion efficiency. The classical understanding suggests that reducing membrane thickness (i.e., shortening the nanochannel length) effectively lowers ion transport resistance, increases ion flux, and thus enhances the achievable power density [122, 127–130].

**Fig. 9:**
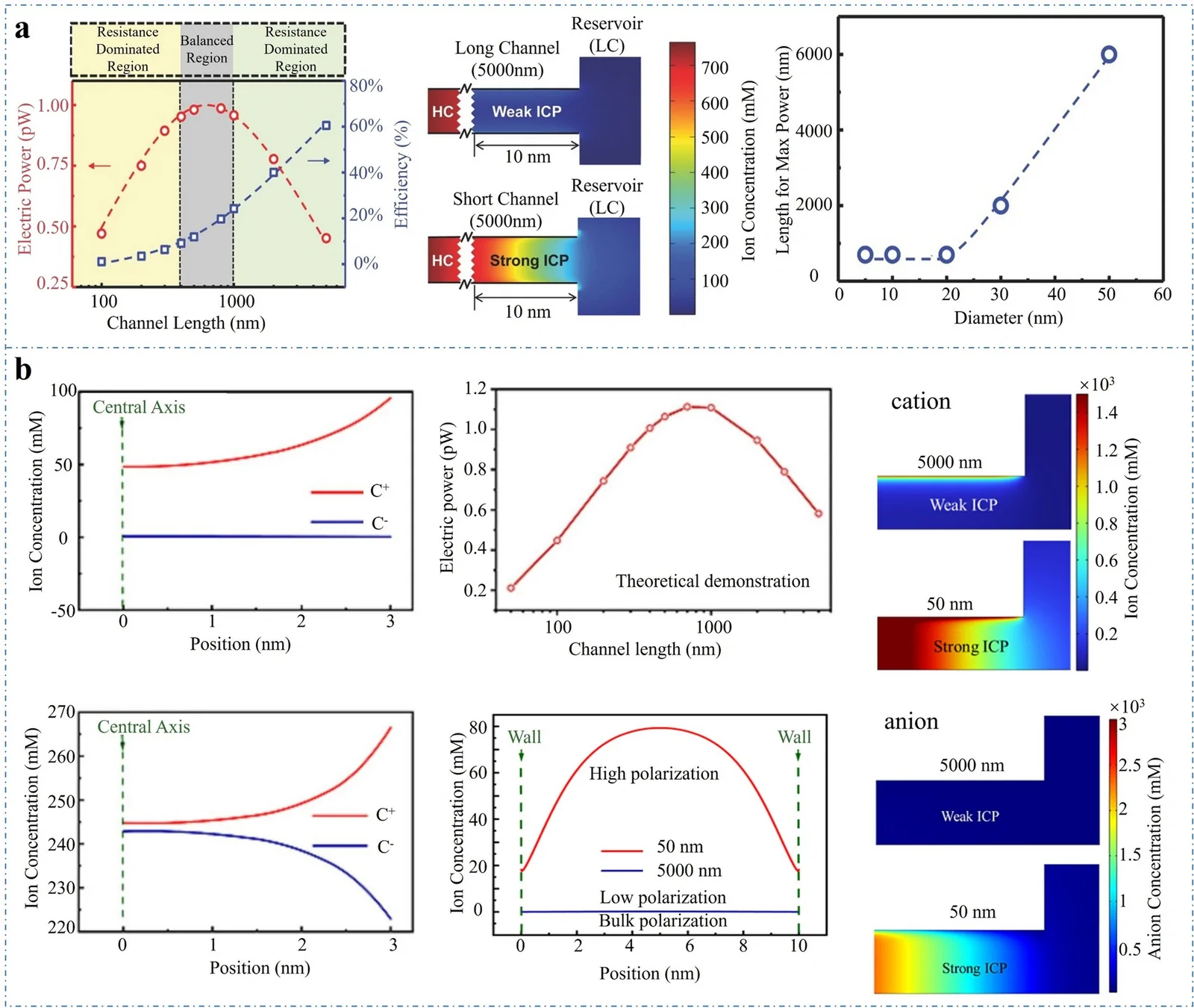
Effect of membrane thickness on osmotic energy conversion. **a** Ion concentration distribution near the low-concentration side of long and short nanochannels and the anomalous channel-length dependence in salinity gradient energy conversion [127]. Copyright © 2017 reprinted by permission of John Wiley and Sons. **b** Numerical simulation of the channel-length dependence during osmotic energy harvesting [25]. Copyright © 2024 reprinted by permission of Elsevier

However, studies have revealed that shorter nanochannels tend to induce more severe ICP. Excessive ICP reduces the ion selectivity of short channels, hinders effective ion transport, and ultimately reduces the salinity gradient energy conversion efficiency (Fig. 9a) [127]. Experimental and theoretical analyses indicate that when the nanochannel length is in the range of 400–1000 nm, a balance between ion selectivity and transport resistance can be achieved (Fig. 9a). When the channel length exceeds 1000 nm, ion transport is mainly governed by the resistance of the nanochannels. In this case, the output power decreases with increasing channel length, showing a resistance-dominated regime similar to an Ohmic response. Conversely, when the channel length is shorter than 400 nm, the system enters a “polarization-controlled regime”, in which the ICP effect is significantly enhanced, and the power output is likewise suppressed. These observations collectively suggest an optimal membrane thickness range for efficient osmotic energy conversion. Moreover, a pronounced synergistic effect between membrane thickness and pore size is observed. As shown in Fig. 9a, when the pore size is small (d < 20 nm), the optimal channel length is ≈ 700 nm. In contrast, for membranes with larger pores (d > 20 nm), a slightly longer channel length is required to sustain sufficient Debye overlap and maintain ion-sieving performance [127].

In other work, Shi et al. [25] demonstrated that tailoring the thickness of cellulose membrane can manipulate the ion transport behavior, thereby optimizing overall membrane performance. By investing, in theory, in the ion concentration distributions at different channel lengths (Fig. 9b), the results showed a significant difference in ion distribution at the low-concentration side when the channel length changed from 5 µm to 50 nm. Compared with short nanochannels, long channels exhibited stronger ion selectivity at the low-concentration side. By coupling ion transport selectivity and distance/length, this finding provides important theoretical guidance for synergistic regulation of thickness and channel structure toward high-performance salinity-gradient energy harvesting.

### Heterostructures

In a salinity-gradient energy conversion system, ICP, arising from disparities in ionic mobilities, represents a major intrinsic bottleneck restricting energy conversion efficiency [124]. This phenomenon typically occurs at the inlet or outlet of the membrane channel, where the mismatch in cation and anion migration rates leads to local concentration inversion or attenuation of the effective salinity gradient, thereby suppressing the establishment of transmembrane potential and current output. In recent years, the rational construction of heterogeneous membranes with well-designed structural and charge asymmetry has emerged as an effective strategy to mitigate ICP, enhance ion rectification, and boost osmotic energy conversion efficiency by enabling directional ion transport and breaking symmetry constraint [131–134].

**Fig. 10:**
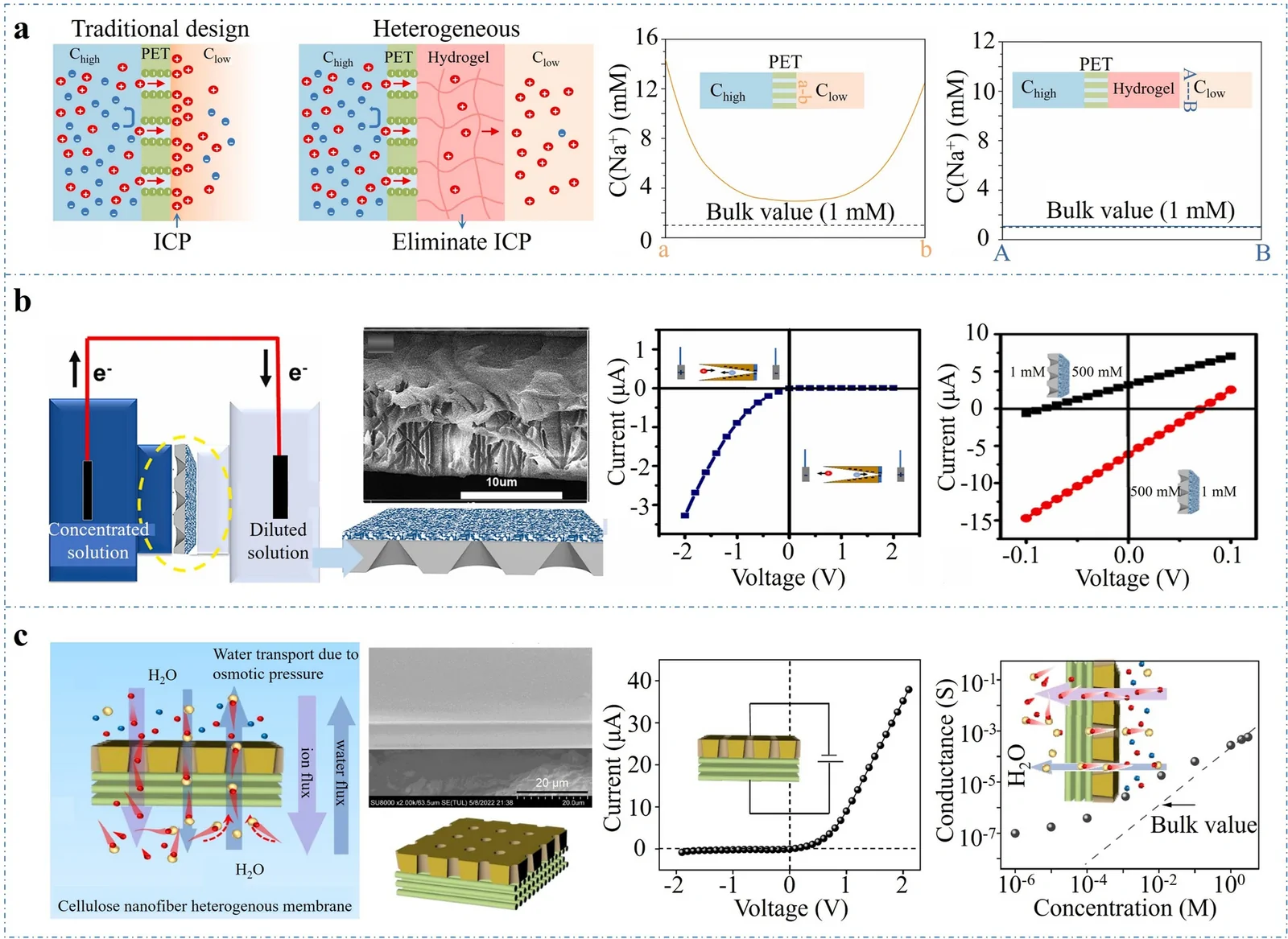
Heterogeneous structure of nanofluidic membrane. **a** Compared with conventional porous membranes, heterogeneous membranes can significantly reduce concentration polarization [140]. Copyright © 2024 reprinted by permission of Elsevier. **b** Osmotic energy conversion performance of charge/structure-heterogeneous membranes [147]. Copyright © 2020 reprinted by permission of Elsevier. **c** Ion transport behavior of asymmetric CNF membranes [46]. Copyright © 2024 reprinted by permission of Springer Nature

Heterogeneous membranes are typically composed of two or more functional layers that exhibit distinct differences in chemical composition, pore architecture, and surface charge density, thereby creating direction-dependent ion transport pathways and breaking transport symmetry. Inspired by the unidirectional ion transport observed in biological ion channels (e.g., the electrocyte structures of electric eels), researchers have developed various biomimetic “artificial ionic diode” membrane architectures [135–137]. The key feature of these structures lies in providing a highly selective or highly charged surface layer on one side, combined with a high-flux layer on the other, thereby inducing ion rectification behavior, promoting directional ion transport, and effectively suppressing back diffusion and local charge accumulation (Fig. 10a) [137–141].

Representative heterogeneous membrane materials include structural gradient/asymmetry [142], opposite charged [140, 143], and coupled them [44]. For example, CNF/metal–organic framework (MOF, e.g., ZIF-8, UiO-66) composite membrane constructs a double-layer structure, including the relative large-pore CNF layer and small-pore MOF, enabling directional Na^+^ transport and significantly enhancing power output [125, 144–146]. For example, Xu et al. [147] employed vacuum filtration to assemble a heterogeneous nanofluidic membrane by integrating carboxylated CNFs (TOCNs) with porous PET substrate, thereby introducing dual asymmetry in surface charge and channel geometry. This configuration effectively mitigated ICP, achieving a remarkable ICR ratio of 562 (71-fold higher than PET membrane) and delivering a power density of 0.96 W m^−2^ under a 50-fold salinity gradient (0.01/0.5 M NaCl) (Fig. 10b). Similarly, Hou et al. [46] fabricated a composite membrane with asymmetric charge distribution, wettability, and structure by coating sulfonated polysulfone (SPSf) onto the sulfonated CNF. Under simulated river water/seawater conditions, this membrane achieved a power density of 8.3 W m^−2^, with conductivity and power density enhanced by 325.0% and 48.2%, respectively (Fig. 10c). These enhancements are attributed to the synergistic action of multiple mechanisms, including electrostatic adsorption, electric double-layer regulation, and geometric rectification, providing new insights for constructing highly efficient ion transport channels [34, 148]. In addition to conventional bilayer or multilayer asymmetric structures, molecular co-assembly has gradually emerged as a simple yet effective strategy for constructing heterogeneous ionic transport environments [149, 150]. For example, ternary molecular co-assembly systems can simultaneously establish charge gradients and interaction gradients while also imparting antibiofouling capability, thereby offering a molecularly programmable pathway for long-term stable osmotic energy conversion in complex aqueous environments [149].

In summary, heterogeneous membrane architectures, by establishing directional structural gradients and charge asymmetry, can simultaneously mitigate ICP, enhance ion selectivity, and improve rectification efficiency, representing a promising design paradigm for high-performance salinity-gradient power generation. Future efforts should focus on improving interfacial stability between heterogeneous layers, maintaining continuous ion-transport pathways, and developing scalable fabrication methods, thereby ensuring long-term operational stability and facilitating practical implementation under complex environmental conditions toward sustainable blue-energy applications.

## Performance of Cellulose Nanofluidic Membrane

The performance of nanofluidic membranes in salinity-gradient systems can be assessed along four main dimensions [151–153]: (1) energy-harvesting capability (efficiency, power density, voltage, current), (2) long-term working stability, (3) mechanical strength, and (4) scalability and cost. Together, these metrics determine whether cellulose-based membranes can move beyond lab-scale demonstrations to industrially relevant blue-energy applications.

### Energy Harvesting Capability

The energy-harvesting capability of a membrane is a key performance indicator that reflects its efficiency in converting salinity-gradient energy into electrical energy. It directly determines the membrane’s potential for practical salinity-gradient power [154, 155]. This capability is primarily characterized by four key parameters, namely, energy conversion efficiency, power density, output voltage, and output current, which represent different dimensions of performance while being interrelated and collectively determining the overall behavior of the membrane during the energy conversion process.

Energy-conversion efficiency is a fundamental parameter that measures the proportion of chemical potential energy converted into electrical energy, reflecting the degree of energy utilization [156, 157]. This efficiency is primarily influenced by factors such as membrane ion selectivity, surface charge density, pore-size distribution, and the magnitude of the salinity gradient [158–160]. From a practical perspective, in systems such as river-seawater interfaces or industrial brine-freshwater gradients, low energy-harvesting efficiency leads to higher electricity cost per unit, oversized system configurations, and reduced energy-return ratios, which limit its economic competitiveness compared to solar and wind energy. Existing studies indicate that, to ensure cost competitiveness, the power density of the membrane should reach at least 5–10 W m^−2^ and the energy conversion efficiency should exceed 30% [79, 161]. Consequently, extensive research has focused on enhancing this parameter through material and structural innovations. For example, He et al. [45] improved the cation selectivity (0.88) of a cellulose-based membrane via surface functionalization, achieving an energy conversion efficiency of 38.3%, which paves the way for advanced energy-harvesting devices. Ren et al. [150] employed a supramolecular assembly strategy to construct ordered ionic channels, achieving an outstanding power density of 21.7 W m^−2^ and an energy conversion efficiency of 46.0%. Notably, in practical salinity gradient energy harvesting systems, the low-concentration side often dominates the overall internal resistance of the system due to the strong dependence of ionic conductivity on solution concentration [162, 163]. When ion mobility in the low-salinity solution becomes limited, both the effective ion flux and conductivity decrease significantly, thereby constraining the overall energy output. In addition, ICP is prone to occur near the channel inlet or outlet, which weakens the effective concentration gradient across the membrane and further reduces the ion migration driving force and energy conversion efficiency [13, 109, 164, 165]. In laminar nanofluidic channels, this phenomenon also introduces a typical design trade-off: enhancing ion selectivity generally requires stronger spatial confinement or higher surface charge density, but this often increases ion transport resistance and exacerbates polarization effects, thereby limiting the net power output of the system [13, 109, 166]. To mitigate these limitations, recent studies have gradually shifted from single-parameter optimization toward systematic regulation of ion transport behavior. By precisely tuning the membrane surface charge distribution, interfacial properties, and ion transport channel structures, it is possible to reduce transport resistance while maintaining high ion selectivity. For example, constructing low-friction or slip interfaces [167] can reduce interfacial resistance and facilitate ion migration. Molecular-scale surface functionalization [168] helps optimize the interfacial charge environment and regulate ion sieving behavior, and the hierarchical porous structures [169] can enhance ion flux while alleviating concentration polarization. These strategies effectively suppress reverse ion diffusion and reduce energy losses, thereby improving the overall salinity gradient energy conversion efficiency [157, 164, 170].

Power density is another key performance parameter for evaluating the practical energy output capability of membranes in salinity-gradient power generation [171–173]. It is defined as the electrical power generated per unit membrane area and is typically determined by multiplying the output voltage and current. Power density intuitively reflects the rate at which the membrane converts the chemical potential energy associated with ion migration into electrical energy, serving as a key indicator for assessing the membrane performance and engineering feasibility [174–176]. Multiple interrelated factors, including ion-migration rate and selectivity, surface charge density, membrane thickness and internal resistance, pore size and porosity, and the electrolyte concentration gradient, govern the magnitude of power density. Insufficient ion selectivity or excessive internal resistance can restrict the net ion flux and cause energy loss, thereby reducing the overall power output [176, 177]. To achieve high power density, the transport performance of the membrane can be optimized through structural and interfacial regulation. On the one hand, reducing membrane thickness, increasing the density of effective transport channels, or constructing three-dimensional porous structures for enhanced ion accessibility can shorten ion-migration pathways and mitigate concentration-polarization effects. On the other hand, tuning surface charge density or designing gradient composite structures strengthens ion-migration driving forces and suppresses backward ion diffusion, thereby synergistically enhancing both voltage and current output [90, 119, 178]. For example, Luo et al. [44] fabricated a cellulose nanofiber membrane that achieved a power density of 7.67 W m^−2^ while maintaining high ion flux. Zhang et al. [179] developed an anti-swelling hydrogel membrane with a power density of 10.1 W m^−2^, which is twice that of the commercial benchmark. These studies demonstrate that the synergistic optimization of micro/nanostructures design and charge distribution regulation provides an effective approach to enhancing membrane power density and overall energy-harvesting capability.

The output voltage refers to the potential difference generated across the membrane arising from the salinity gradient, which acts as the fundamental electrochemical driving force for ion migration and current generation [180, 181]. The magnitude of the output voltage primarily depends on the salinity gradient, the fixed charge density of the membrane, and its surface electrostatic potential characteristics [161, 182]. A larger concentration gradient provides a higher chemical potential difference, thereby increasing the theoretical voltage output. The fixed charge density governs the ion-selective transport at the membrane interface; a higher charge density promotes the enrichment of counterions and intensifies the internal electric field across the nanochannels. Meanwhile, the surface potential characteristics of the membrane (such as zeta potential and uniformity of surface charge distribution) determine the potential difference at the interface, further influencing the output voltage [183]. Current strategies to enhance voltage include increasing the fixed-charge density of the membrane, constructing potential-gradient structures, and adopting multilayer composite membrane designs [158, 184, 185]. For example, Chen et al. [185] fabricated a biomimetic GO/ANF composite membrane that achieved an Voc of approximately 1.61 V under simulated seawater/freshwater conditions by connecting multiple membrane units in series, sufficient to power small electronic devices. Similarly, Wang et al. [158] developed a cellulose nanofiber membrane with both positive and negative surface charges, where tuning the charge density and connecting multiple membrane pairs in series resulted in an output voltage of 3.11 V. These findings demonstrate that optimizing the charge characteristics and structural design of membranes can effectively enhance voltage output, providing crucial support for efficient salinity-gradient energy conversion.

Current refers to the number of ions passing through the membrane per unit time, directly reflecting the membrane’s ion-transport capability and serving as a key parameter that determines the actual electrical output of a salinity-gradient power generation system [186, 187]. The current density, together with the output voltage, jointly determines the instantaneous power of the membrane. Its magnitude is influenced by multiple factors, including membrane porosity and pore-size distribution, thickness and internal resistance, surface charge density and ion selectivity, as well as the salt concentration gradient under operating conditions [188, 189]. For cellulose-based membranes, the highly ordered nanochannels formed by the alignment of nanofibers can significantly enhance ion migration rates, while surface functionalization (such as the synergistic distribution of positive and negative charges) can improve target ion selectivity and suppress reverse ion diffusion, thereby effectively increasing the net current output [47, 190]. Strategies for strengthening current output include increasing the number of effective ion channels, reducing membrane thickness, regulating surface charge, and constructing three-dimensional nanochannel networks to shorten ion migration pathways and reduce concentration polarization [156, 191–193]. For example, Wu et al. [21] developed a wood-derived cellulose nanofiber membrane that retained aligned nanochannels, and underwent surface functionalization; its ion transport capability was significantly improved. Under a 1000-fold concentration gradient, the developed wood membrane achieved a current density of 286.3 mA m^−2^. These studies indicate that the synergistic optimization of intrinsic material properties and micro-/nanostructural design, particularly through the application of cellulose nanofiber membranes, can effectively accelerate ion migration, thereby enhancing the overall energy-harvesting capability of salinity-gradient energy-conversion systems.

In summary, the energy-harvesting capability of a membrane is jointly determined by multiple parameters, including energy conversion efficiency, power density, voltage, and current. These parameters are strongly interrelated and are influenced by both the intrinsic properties of the membrane material and the regulation of its micro/nanostructure. Through the synergistic optimization of surface charge distribution, pore structure configuration, and functional modification, simultaneous enhancement of multiple parameters can be achieved, thereby significantly improving the overall performance and application potential of the membrane in salinity-gradient power generation systems.

### Long-Term Working

Long-term working stability is defined as the capability of membrane materials to maintain their structural integrity and functional performance over extended periods under complex aqueous, chemical, and biological environments [194, 195]. It serves as a critical indicator for assessing their potential for sustainable operation and large-scale engineering applications in salinity-gradient energy conversion systems. During operation, cellulose-based membranes often face multiple challenges, including water intrusion, salt ion corrosion, and microbial contamination or enzymatic degradation. These factors can result in deteriorated ion-transport performance, progressive voltage decay, and even eventual system failure [41, 196–199]. Therefore, investigating the stability of membranes under the influence of water, salt ions, and microorganisms is of great significance for achieving long-term, high-efficiency power generation.

Cellulose molecules contain a large number of hydrophilic hydroxyl groups (-OH), which make them prone to water absorption, swelling, or hydration reactions in aqueous environments, leading to membrane structural relaxation, pore size changes, and decreased ion selectivity [200, 201]. Under high humidity or prolonged immersion, water molecules can also induce chain scission and damage to the microscopic pore structure, thereby reducing the mechanical strength and electrochemical stability of the membrane [202–204]. To enhance hydrolytic stability and anti-swelling resistance, chemical crosslinking treatment is commonly employed as an effective reinforcement strategy. By introducing crosslinkers such as glutaraldehyde, epichlorohydrin, or isocyanates, a dense three-dimensional network is formed within the membrane, effectively restricting molecular chain mobility [205–207]. For example, Yang et al. [47] utilized the carboxyl groups of 1, 2, 3, 4-Butanetetracarboxylic acid to chemically crosslink with the hydroxyl groups on cellulose chains, introducing abundant negative charges on the membrane surface. This modification enhanced the cation-selective transport capability of the nanocellulose membrane. Even after continuous operation for 12 h under pH 3–9 conditions, the membrane maintained excellent ion selectivity and structural stability. In recent years, research on anti-swelling strategies has evolved from single crosslinking approaches toward multiscale network reinforcement and dynamic structural stabilization [208, 209]. In terms of material design, green multifunctional crosslinking systems have been constructed using bio-based crosslinkers such as citric acid or metal-coordination bonds, which suppress swelling while simultaneously enhancing toughness capability [210–213]. In terms of structural regulation, interpenetrating polymer networks [208, 214] have been developed, or high-aspect-ratio nanofillers such as graphene oxide and cellulose nanocrystals have been introduced to pin cellulose chains at the microscale, thereby further reducing water uptake and improving structural stability [209, 215–220]. In terms of interfacial engineering, hydrophobic chain segments have been grafted onto the surface or ultrathin dense layers have been constructed to form physical barriers that hinder water molecule penetration [221–224]. These multiscale strategies not only markedly improve the dimensional stability and mechanical durability of cellulose membranes in aqueous environments [209, 218, 222], but also enable the integration of anti-swelling behavior with high ion selectivity and antifouling performance, thus providing new insights for their application in osmotic energy harvesting [47, 138, 225, 226].

In high-salinity environments (e.g., seawater or brine), highly concentrated ions such as Cl^−^, SO_4_^2−^, and Na^+^ interact with the charged functional groups of membranes, such as hydroxyl, carboxyl, and sulfonic moieties, through ion shielding, ion exchange, and oxidative or substitutional degradation reactions [156, 227–229]. These interactions reduce the membrane’s fixed charge density and disrupt its charge repulsion effect, leading to decreased ion selectivity, deteriorated conductivity, and lower power generation efficiency. In addition, some corrosive ions, such as Cl^−^, can promote oxidative reactions, accelerating the chemical degradation of the membrane. To resist such effects, researchers have employed strategies such as hydrophobization [230, 231] (e.g., esterification or silanization), introduction of antioxidant functional groups [232], or hybridization with inorganic nanoparticles [233–235] (e.g., nano-SiO_2_, TiO_2_, and GO) to enhance chemical stability. For example, Song et al. [57] used a one-step microbial cultivation method combined with GO to construct a bacterial cellulose membrane featuring 1D/2D nanochannel structures. Under a 500-fold salinity gradient (artificial seawater/river water system), the membrane achieved an energy conversion efficiency of approximately 31.4% and a power density of 7.49 W m^−2^. After 20 days of continuous operation, the power output decreased by only 4.4%, demonstrating excellent long-term stability in high-salinity environments.

In natural seawater or wastewater environments, membrane surfaces are inevitably susceptible to the adsorption of organic matter and the subsequent microbial colonization, leading to biofilm formation. Such fouling may result in severe blockage of ion transport channels and partial neutralization of surface functional charges [236, 237]. Under prolonged immersion, cellulases secreted by microorganisms can enzymatically degrade the cellulose backbone, thereby damaging the membrane structure and deteriorating its energy conversion performance [225, 238]. To address these challenges, both surface and bulk antimicrobial/antifouling modification strategies have been demonstrated to be effective. For example, incorporating silver ions, quaternary ammonium groups, or TiO_2_ nanoparticles into cellulose membranes can effectively inhibit microbial adhesion, biofilm formation, and enzymatic biodegradation, thereby enhancing their long-term stability in complex aqueous environments [138, 239–241]. For example, Li et al. [242] demonstrated that uniformly dispersing nanosized Ag/TiO_2_ particles within a cellulose scaffold can significantly inhibit bacterial growth, thereby imparting excellent antibacterial properties to the material. In addition, Liu et al. [243] utilized the broad-spectrum antibacterial activity of quaternized chitosan (Q-CS) to construct a composite membrane with outstanding anti-biofouling performance, which maintained stable antibacterial efficacy for up to 25 days. In addition, by creating a highly hydrophilic double-network structure, the formation of a physical barrier via the hydration layer can effectively inhibit microbial adhesion [239, 244, 245]. Sun et al. [239] introduced an acrylic acid-acrylamide-methyl acrylate copolymer network into a BC matrix to fabricate a double-network hydrogel membrane. When applied to salinity-gradient power generation in a pulp black liquor/water or seawater mixture, the membrane achieved a power density of 28.4 W m^−2^. Moreover, the membrane maintained high power density stably under the black liquor/seawater gradient for up to 14 days, demonstrating excellent long-term stability and robust performance in complex water environments.

Overall, water-induced swelling, ion-induced chemical degradation, and microbial biodegradation are the three predominant factors governing the long-term operational stability of cellulose-based membranes. By synergistically implementing strategies such as structural crosslinking, surface or interfacial modification, inorganic hybridization, and antimicrobial functionalization, the chemical and biological stability of membranes in high-salinity, high-humidity, and microbially active environments can be significantly enhanced. This, in turn, prolongs the service lifespan and ensures sustained energy conversion efficiency, operational reliability, and economic viability of salinity-gradient power generation systems.

### Mechanical Strength

Mechanical strength is the ability of membrane materials to resist mechanical deformation and structural failure under applied external forces, such as tension, compression, shear, or osmotic pressure. It is a critical parameter for evaluating whether a membrane can operate stably and reliably in osmotically driven energy conversion systems [96, 246–249]. In practical salinity-gradient power generation, membranes are subjected to high pressure differentials, elevated flow velocities, and frequent on–off operation cycles. Insufficient mechanical strength may result in deformation, interlayer delamination, or rupture, thereby compromising system continuity and operational safety [239, 248, 250–252]. In addition, high mechanical strength is also required during membrane fabrication, drying, and device assembly to prevent cracking or collapse, ensuring process operability and structural integrity. Therefore, cellulose-based membrane must exhibit robust mechanical performance under both dry and wet conditions to meet the full-cycle requirements from manufacturing to practical operation in real-world energy conversion systems [47, 251, 253, 254].

Dry strength refers to the ability of cellulose-based membranes to resist stretching, tearing, or breaking in a dry state. It reflects the membrane’s ability to maintain structural integrity during fabrication, film forming, and device assembly, and thus indicates its process stability [176, 248, 255, 256]. Dry strength primarily depends on the hydrogen bonding between cellulose molecular chains, the content of crystalline regions, and the molecular alignment. When the molecular chains are well-ordered and crystallinity is high, a dense hydrogen-bond network is established within the membrane matrix, thereby improving load transfer efficiency and structural stability [31, 47, 257, 258]. To enhance dry strength, researchers commonly use strategies such as nano-reinforcement, chemical crosslinking, and structural alignment [47, 259–261]. For example, CNF or CNCs can form dense hydrogen-bonded networks, significantly increasing the tensile modulus and fracture strength [31, 262]. Chemical crosslinking with glutaraldehyde, epichlorohydrin, or isocyanates can create a stable three-dimensional covalent network, thereby improving deformation resistance [263, 264]. For example, Gao et al. [265] employed a glutaraldehyde (GA)-assisted crosslinking strategy to fabricate crosslinked regenerated cellulose films, resulting in a significant increase in tensile strength (240.9% higher than that of RC films). In addition, controlling the alignment of CNFs can produce crystalline ordered structures, further enhancing the mechanical performance of dry membranes [266–268]. Yang et al. [267] fabricated unidirectionally aligned CNF films using a pressure-controlled extrusion process, achieving a tensile strength of up to 245 MPa.

Wet strength refers to the ability of a membrane to resist external forces in damp or soaked conditions. It serves as a key parameter for assessing the structural stability and long-term reliability of membranes in salinity-gradient energy conversion systems [31, 62, 247, 269]. Since cellulose molecules contain abundant hydrophilic hydroxyl groups (-OH), hydrogen-bond competition occurs between water molecules and cellulose chains under humid or aqueous conditions, thereby weakening the original intermolecular interactions [270, 271]. Consequently, the membrane undergoes swelling, pore enlargement, and deteriorated mechanical integrity. Under saline conditions, ion shielding and osmotic effects can further disrupt the hydrogen-bond network, thereby reducing wet strength to a level substantially lower than its dry counterpart. Wet strength retention is governed by multiple factors, including cellulose hydrophilicity, crosslinking density, porosity, and the incorporation of inorganic reinforcing phases. To mitigate these effects and enhance wet strength, common strategies include chemical crosslinking, inorganic hybridization, and surface hydrophobization [271–273]. Introducing crosslinkers such as glutaraldehyde, epoxy compounds, or isocyanates can restrict molecular chain mobility and improve hydrolytic and dimensional stability [47, 274]. Incorporating inorganic nanoparticles such as SiO_2_, TiO_2_, or GO can reinforce the membrane framework and effectively suppress swelling [31, 275, 276]. Surface modifications through esterification or silanization can lower surface hydrophilicity, hindering water penetration and plasticization [257, 277–279]. Zhang et al. [279] modified pretreated bamboo powder via a one-step maleic anhydride (MAH) esterification process to prepare maleic anhydride-esterified bamboo-based nanofibers (MLCNF). After esterification, the wet tensile strength of MLCNF increased to 43.5 MPa, more than six times that of unmodified LCNF. In addition, multiscale synergistic reinforcement strategies can significantly enhance the wet mechanical properties of cellulose membranes, providing a solid foundation for their long-term stable operation in complex saline and aqueous environments [49, 274, 280–283]. Zhou et al. [49] constructed a regenerated cellulose nanofluidic network that exhibited a wet strength of 29 MPa and stable operation for 43 days in an artificial seawater/river water system, demonstrating excellent durability.

In summary, dry strength governs the processing stability of membranes during fabrication and assembly, whereas wet strength dictates their operational reliability under complex aqueous and saline conditions. By implementing multiscale synergistic reinforcement strategies, including nanoreinforcement, chemical crosslinking, structural alignment, and surface hydrophobization, the mechanical performance of cellulose-based membranes can be simultaneously enhanced under both dry and wet conditions, thereby ensuring long-term operational stability and high energy conversion efficiency in salinity-gradient systems.

### Scalability and Low Cost

Scalability and low cost are key criteria for evaluating the industrial viability of membrane materials, which directly determine the feasibility of salinity-gradient energy conversion systems in engineering-scale and commercial applications. Specifically, scalability requires that membrane materials can be produced via cost-effective and scalable fabrication routes while ensuring consistent performance and quality. Meanwhile, low cost is primarily reflected in economic advantages associated with raw material sourcing, energy consumption, processing complexity, and equipment investment [192, 284–286]. However, if membrane fabrication involves high energy consumption, costly raw materials, or poor scalability due to complex processing routes, even membranes exhibiting excellent electrochemical performance at the laboratory scale are unlikely to meet the demands for long-term operational stability and real-world industrial deployment.

Cellulose-based membranes are derived from renewable biomass resources (e.g., plant fibers and agricultural residues such as straw, cotton linters, and bagasse), offering abundant availability, low cost, and inherent sustainability [287, 288]. CNF or CNC can be produced via mechanical fibrillation, enzymatic hydrolysis, or mild chemical oxidation, which can subsequently be assembled into membrane architectures, thereby demonstrating the scalability while maintaining desirable functional performance [289, 290]. In addition, cellulose-based membranes exhibit distinct advantages in fabrication versatility, offering compatibility across multiple scalable processing routes. Specifically, various green and energy-efficient fabrication methods have been developed, including electrospinning, self-assembly, template-assisted deposition, and layer-by-layer assembly, which demonstrate excellent process compatibility and scalability [45, 91, 291]. For example, Shi et al. [25] prepared CMA membranes via cellulose molecular self-assembly, achieving a power density of 2.27 W m^−2^ and a maximum voltage of 1.32 V, with continuous operation for over 100 days, indicating excellent stability and scalability. Li et al. [292] prepared phosphorylated cellulose membrane via a urea-assisted dehydration reaction, achieving power densities of 6.42 W m^−2^ under a 50-fold salinity gradient and 22.1 W m^−2^ under hypersaline conditions. A tandem stack of 24 units generated an output voltage of 1.8 V, successfully powering an electronic calculator.

Although cellulose-based membranes have demonstrated excellent ion selectivity and high power density in laboratory studies, their practical translation to operational RED systems remains a significant challenge [12, 293, 294]. Beyond their superior electrochemical performance, practical deployment of RED systems requires membranes that can be fabricated at scale, with precisely controlled thickness and porosity, uniform and stable surface-charge distribution, and sufficient mechanical integrity to withstand stack compression and prolonged operation under high-salinity gradients [50, 158, 293, 295]. In addition, membranes must possess chemical stability and fouling resistance under realistic feedwater conditions, which may contain multivalent ions, organic contaminants, and microorganisms. In recent years, efforts have been made to explore the engineering feasibility of cellulose-based membranes. For membrane fabrication, continuous manufacturing strategies such as roll-to-roll processing offer potential pathways for large-area, low-cost production. At the same time, some studies have demonstrated the preliminary integration of cellulose-based membranes into multi-unit RED stacks, achieving output voltages sufficient to power small electronic devices [29, 158, 293, 296]. These advances indicate the potential of renewable biomass-derived membranes for sustainable salinity-gradient energy harvesting. Nevertheless, several critical engineering challenges must be addressed to enable large-scale RED deployment. First, membrane resistance needs to be further reduced while maintaining high ion selectivity to improve the overall energy conversion efficiency of RED systems. Second, the geometries of membranes and spacers should be optimized to minimize concentration polarization and hydraulic losses, thereby enhancing system power output. Finally, the structural and chemical stability of membranes under long-term operation must be further improved to ensure continuous and reliable energy generation. It is noteworthy that industrial-scale RED systems typically require power densities exceeding 5 W m^−2^ under realistic salinity gradients [79], placing stringent demands on membrane ion transport, mechanical stability, and fouling resistance. Therefore, future research should integrate scalable membrane fabrication with stack-level optimization, and adopt a system-level approach that coordinates the design of membranes, spacers, and electrodes to enhance energy conversion efficiency and operational stability. Addressing these key challenges will be essential to advance cellulose-based membranes from laboratory prototypes toward practical RED applications [12, 293, 294, 297, 298].

It should also be emphasized that the sustainability of cellulose-based membranes cannot be taken for granted simply because cellulose itself is renewable and biodegradable [299–303]. When cellulose is converted into extensive functionalization, the associated processing steps, such as intensive mechanical disintegration, harsh drying, and chemical pretreatment or modification, may introduce non-negligible environmental burdens in terms of energy demand, water consumption, and chemical toxicity [300–303]. Therefore, future development should follow a “sustainability-by-design” principle [299–301]. This includes: (1) prioritizing low-toxicity and recyclable solvent systems while minimizing salt- and acid-containing waste streams [304, 305], (2) reducing unit-operation intensity by integrating pretreatment, fibrillation, and assembly steps [299, 303], (3) making greater use of biomass residues and mild enzymatic or oxidative approaches wherever possible [300, 301, 306], and (4) benchmarking advanced membrane systems by combining electrochemical metrics with life cycle assessment (LCA) and techno-economic analysis (TEA) [304, 305, 307]. Such a balanced framework is essential to ensure that the intrinsic green advantages of cellulose are genuinely translated into sustainable blue-energy technologies [299–301, 307].

In summary, cellulose-based membranes, derived from renewable and low-carbon feedstocks and fabricated through green and highly tunable processes, exhibit strong potential for scalable deployment in sustainable salinity-gradient energy conversion systems. Future research should integrate efficient utilization of renewable resources, green manufacturing technologies, and industrial-chain integration to facilitate the translation of cellulose-based membranes from laboratory research toward scalable commercial production and engineering implementation.

## Conclusion and Perspective

Cellulose-based materials have shown strong potential for osmotic energy conversion technologies, particularly for sustainable blue energy harvesting. As a natural and renewable biopolymer, cellulose combines outstanding mechanical robustness, chemical tunability, ion selectivity, and biodegradability, making it a promising material platform for osmotic energy conversion systems. Through targeted chemical modification, structural nanocomposite engineering, and interfacial functionalization, the ionic conductivity, mechanical strength, fouling resistance, and stability of cellulose membranes have been significantly improved. These advances collectively contribute to enhanced energy conversion efficiency and extended operational lifespan of cellulose-based osmotic membranes.

Although cellulose-based membranes offer clear advantages over conventional petroleum-derived polymer membranes (e.g., Nafion, polyamides) in terms of high conversion performance, ease of manufacturing, and environmental sustainability, they still encounter several obstacles when translated to practical or industrial-scale osmotic energy systems. The key challenges are ensuring long-term operational stability, especially in aqueous environments, further improving energy-harvesting efficiency, and enhancing overall techno-economic feasibility. These limitations currently impede the commercial deployment of cellulose-based membranes in osmotic energy conversion systems. Therefore, further technological innovation and systematic optimization remain essential to facilitate the large-scale implementation of cellulose-based membranes for sustainable blue energy harvesting.

Future research on cellulose-based membranes should emphasize optimizing ion transport dynamics, reinforcing mechanical robustness, and improving antifouling performance, while enabling scalable fabrication and industrial-level implementation. To overcome the challenges mentioned above, future efforts should be directed toward the following aspects: (1) Ensuring long-term stability: Reinforce the chemical and structural durability of membranes under harsh saline and variable thermal conditions via chemical crosslinking, functional modification, or nanocomposite reinforcement. Particular attention should be paid to achieving uniformity and reproducibility in large-area fabrication. (2) Enhancing antifouling performance: Develop antifouling surface coatings or hierarchical porous architectures to mitigate foulant adhesion, maintain ion transport efficiency, and extend membrane service lifespan. (3) Lowering fabrication costs and environmental footprint: Utilize biogenic residues and industrial by-products as renewable feedstocks, combined with green, energy-efficient fabrication routes, to enable scalable and cost-effective production. (4) Rational design and functional optimization: Enhance ion selectivity and conductivity via surface functionalization, nanocomposite engineering, and structural tuning; integrate multi-scale theoretical modeling to establish structure–property correlations and guide performance optimization. (5) Broadening application horizons: Explore emerging fields such as nanofluidic systems, microfluidic electronics, and biosensing or health-monitoring platforms, aiming to integrate multifunctionality with efficient energy conversion. Through interdisciplinary collaboration and continuous innovation, cellulose-based membranes are expected to evolve into key materials for next-generation sustainable osmotic energy systems and multifunctional nanofluidic platforms, thereby bridging the gap between laboratory research and large-scale industrial application.

## Abbreviations

RC: Regenerated cellulose
BC: Bacterial cellulose
OC: Oxidized cellulose
RED: Reverse electrodialysis
PRO: Pressure-retarded osmosis
Voc: Open-circuit voltage
I_sc_: Short-circuit current
E_m_: Output voltage
I_osm_: Osmotic current
CNFs: Cellulose nanofibers
CNCs: Cellulose nanocrystals
EDL: Electric double layer
ICR: Ion current rectification
ICP: Ion concentration polarization
CEMs: Cation exchange membranes
AEMs: Anion exchange membranes
CNT: Carbon nanotubes
COF: Covalent organic framework
MOF: Metal–organic framework
GO: Graphene oxide
LDCM: Low-dimensional carbon material
2D: Two-dimensional
